# DeepInterAware: Deep Interaction Interface‐Aware Network for Improving Antigen‐Antibody Interaction Prediction from Sequence Data

**DOI:** 10.1002/advs.202412533

**Published:** 2025-02-11

**Authors:** Yuhang Xia, Zhiwei Wang, Feng Huang, Zhankun Xiong, Yongkang Wang, Minyao Qiu, Wen Zhang

**Affiliations:** ^1^ College of Informatics Huazhong Agricultural University Wuhan 430070 China

**Keywords:** antigen–antibody interaction, binding free energy change, deep learning, sequence‐based prediction

## Abstract

Identifying interactions between candidate antibodies and target antigens is a key step in developing effective human therapeutics. The antigen–antibody interaction (AAI) occurs at the structural level, but the limited structure data poses a significant challenge. However, recent studies revealed that structural information can be learned from the vast amount of sequence data, indicating that the interaction prediction can benefit from the abundance of antigen and antibody sequences. In this study, **DeepInterAware** (deep interaction interface‐aware network) is proposed, a framework dynamically incorporating interaction interface information directly learned from sequence data, along with the inherent specificity information of the sequences. Experimental results in interaction prediction demonstrate that DeepInterAware outperforms existing methods and exhibits promising inductive capabilities for predicting interactions involving unseen antigens or antibodies, and transfer capabilities for similar tasks. More notably, DeepInterAware has unique advantages that existing methods lack. First, DeepInterAware can dive into the underlying mechanisms of AAIs, offering the ability to identify potential binding sites. Second, it is proficient in detecting mutations within antigens or antibodies, and can be extended for precise predictions of the binding free energy changes upon mutations. The HER2‐targeting antibody screening experiment further underscores DeepInterAware's exceptional capability in identifying binding antibodies for target antigens, establishing it as an important tool for antibody screening.

## Graphical abstract

Many thanks to you for your efforts on our manuscript. Here is a short summary and an eye‐catching color image of our manuscript. DeepInterAware is a sequence‐based framework that overcomes the challenge of scarce structure data in antigen‐antibody interactions. It outperforms existing methods, and is proficient in identifying binding sites, detecting mutations, and predicting binding energy changes. The HER2‐targeting antibody screening highlights its ability to identify binding antibodies for target antigens, making it a useful tool for antibody screening.

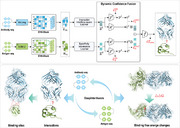

## 1 Introduction

The antigen–antibody interaction (AAI) represents the specific chemical interaction that unfolds between antibodies, generated by B cells, and their target antigens as part of the body's immune response. In this process, antibodies act as vigilant sentries, defending the body by pinpointing and neutralizing pathogenic markers on infected cells and foreign pathogens. This mechanism provides therapeutic interventions^[^ ^1^, ^2^ ^]^ for severe diseases, including cancer, autoimmune conditions, and vaccine development. Identifying AAIs helps to screen antibodies that bind to specific antigens, and is important for developing effective human therapeutics. Traditional laboratory techniques for identifying AAIs, such as the radioimmunoassay^[^ ^3^ ^]^ and enzyme‐linked immunosorbent assay^[^ ^4^ ^]^ are laborious and costly. Consequently, there is a pressing demand for computational methods to streamline the prediction of AAIs, thereby speeding up the discovery of therapeutic antibodies.

In recent years, artificial intelligence techniques such as deep learning have been widely used for AAI prediction, and these methods can be broadly categorized into two major classes: structure‐based and sequence‐based. Structure‐based methods utilize the structure data of antigens and antibodies to build the prediction models. For example, Schneider et al.^[^ ^5^ ^]^ implemented a convolutional neural network (CNN) trained on rapidly generated rigid‐body docking poses of modeled antibody structures in complex with antigen epitopes, and combined ZDOCK docking scores to predict antigen‐antibody (Ag–Ab) binding. Although the utilization of structures can significantly increase the accuracy of AAI prediction methods, the costly and laborious wet lab procedures necessary to procure these structures inevitably restrict the widespread applicability of structure‐based methods. Due to the abundance of available sequence data, a series of sequence‐based prediction methods have been developed. For example, Mason et al.^[^ ^6^ ^]^ utilized a CNN to learn antibody sequences from the trastuzumab mutation library and predicted antibodies with specific binding to HER2. Following the same paradigm, Lim et al.^[^ ^7^ ^]^ trained a CNN to learn sequence features of complementarity‐determining region (CDR) loops for predicting binding specificity to antigens the PD‐1 and CTLA‐4. Huang et al.^[^ ^8^ ^]^ employed a Siamese‐like CNN architecture to learn the CKSAAP (composition of k‐spaced amino acid pairs) features of antigen and antibody sequences for predicting Ag–Ab binding. Zhang et al.^[^ ^9^ ^]^ developed the framework DeepAAI, which utilizes an adaptive relation graph learning to capture global features and employs a CNN to extract local features from sequence data for predicting Ag–Ab neutralization effects. The interactions between antigens and antibodies occurs at the structural level, where the interaction interface, composed of binding sites, provides crucial insights into the underlying mechanisms, but existing sequence‐based methods fail to consider this, limiting predictive accuracy.

Over the past few years, we have witnessed advancements of large language models and their applications in biology. Based on vast number of sequence data, pre‐trained protein language models (such as ESM‐2^[^ ^10^ ^]^ and ProtTrans^[^ ^11^ ^]^) and pre‐trained nucleic acid language models (such as RNAErnie^[^ ^12^ ^]^ and megaDNA^[^ ^13^ ^]^), benefit a variety of prediction tasks, including protein–protein interactions,^[^ ^14^ ^]^ drug–target interactions,^[^ ^15^, ^16^ ^]^ protein–nucleic acid interactions^[^ ^17^ ^]^ and RNA‐RNA interactions.^[^ ^18^ ^]^ Although the scarcity of structure data undermines the development of AAI prediction, several public databases, including UniRef^[^ ^19^ ^]^ and OAS,^[^ ^20^ ^]^ provide plenty of antigen and antibody sequences, and the antibody language models, such as AbLang^[^ ^21^ ^]^ and AntiBERTy,^[^ ^22^ ^]^ have also been developed for the downstream tasks.^[^ ^23^, ^24^ ^]^ More importantly, a recent study^[^ ^25^ ^]^ has shown that leveraging insights from protein and nucleic acid language modeling can enhance the generalizability of AI models for biomolecular interaction prediction, and further revealed that structural information can be derived from a broad set of sequence data. Inspired by these studies, AAI prediction could be significantly improved by leveraging sequence data and pre‐trained language models to learn the implicit structural information of the interactions.

In this paper, we introduce an innovative framework named **DeepInterAware**, which dynamically integrates interaction interface information directly learned from sequence data along with the inherent specificity information of the sequences. Experimental results in interaction prediction demonstrate that DeepInterAware outperforms existing state‐of‐the‐art methods, showcasing its strong inductive capabilities with antigens or antibodies that were previously unseen, along with its capacity to transfer knowledge effectively to similar tasks. Beyond the advantage of AAI prediction accuracy, DeepInterAware offers additional benefits that existing methods lack. First, DeepInterAware could capture and understand the underlying mechanisms of AAIs, equipping it with the ability to identify potential binding sites merely based on sequence data. Furthermore, DeepInterAware is proficient in detecting mutations within antigens or antibodies, and can be further extended to predict the binding free energy changes upon mutations. The HER2‐targeting antibody screening experiment has further highlighted DeepInterAware's outstanding proficiency in pinpointing antibodies that bind to specific antigen targets, establishing it as a useful tool for antibody screening.

## 2 Results

### 2.1 DeepInterAware Framework

**Figure 1:**
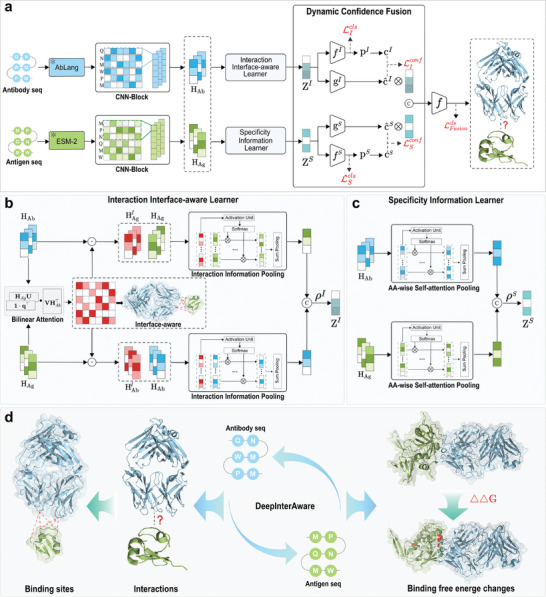
The overview of DeepInterAware. a) The overall framework of DeepInterAware. First, the designed Interaction Interface‐aware Learner (IIL) and Specificity Information Learner (SIL) are employed to learn the amino acid features extracted from the large language model. Subsequently, the Dynamic Confidence Fusion (DCF) module is responsible for dynamically fusing the features obtained by these two learners. Finally, these fused features are passed to the subsequent interaction prediction module to predict AAIs. b) The IIL module captures the interaction interface information between antigens and antibodies. c) The SIL module captures the inherent specificity information of the sequences. d) The applications of DeepInterAware include AAI prediction, binding site identification, and binding free energy change prediction.

As shown in **Figure** **1**a–c, DeepInterAware presents a framework for predicting AAIs from sequence data. DeepInterAware first obtains embeddings of antigen and antibody sequences through the pre‐trained language models ESM‐2 and AbLang respectively, and employs two well‐designed modules: the Interaction Interface‐aware Learner (IIL) and the Specificity Information Learner (SIL), to capture implicit interaction interface information about the AAIs and the inherent specificity information of the antigens (antibodies). The IIL could identify the potential binding sites (epitopes and paratopes) of antigens and antibodies, thereby improving the AAI prediction. Then, we introduced a Dynamic Confidence Fusion (DCF) module to combine the specific information and interaction interface information, which may complement each other, and finally built the prediction model. As shown in Figure 1d, DeepInterAware can predict AAIs, identify binding sites, and predict the binding free energy changes due to mutations.

### 2.2 DeepInterAware Outperforms the State‐of‐the‐Art Methods in Antigen–Antibody Interaction Predction

In this section, we compared the performance of the proposed method, DeepInterAware, against other leading AAI prediction methods, on binding and neutralization tasks. We utilized the AVIDa‐hIL6 and SAbDab datasets for the binding task, and the HIV dataset for the neutralization task. Additionally, we utilized the HIV and CoV‐AbDab datasets to assess the models' transferability to new antigens. Details on the experiment implementation can be found in Section S1.1 (Supporting Information).

The performances of all methods in binding prediction are demonstrated in **Table** **1**. On the AVIDa‐hIL6 dataset, DeepInterAware outperforms baselines in AUPRC and MCC and achieves a competitive result in AUROC. Among them, the AUPRC and MCC are improved by 0.2% and 0.4%, compared with the second‐best methods. The SAbDab dataset is smaller than the AVIDa‐hIL6 dataset but includes more diverse antigens and antibodies with low sequence homology (Figure S1, Supporting Information) and increases the difficulty of binding prediction. Table 1 shows a marked decline of all methods' performances on this dataset. Nonetheless, DeepInterAware achieves improvements over the second‐best methods by 4.1%, 6.9% and 5.2% in AUROC, AUPRC, and MCC, respectively. The superior performance of DeepInterAware is due to its ability to capture both interaction and specificity information of Ag–Ab sequences and acquire general knowledge for the binding prediction across diverse antigens and antibodies.

**Table 1:** Binding performance comparison on AVIDa‐hIL6 and SAbDab datasets.

Dataset	Model	AUROC	AUPRC	MCC	ACC	F1	Precision	Recall
AVIDa‐hIL6	DrugBAN	**0.971 ± 0.016**	0.933 ± 0.027	0.920 ± 0.019	0.995 ± 0.001	0.920 ± 0.020	**0.981 ± 0.010**	0.866 ± 0.033
	ESM2AntiBERTy	0.965 ± 0.014	0.910 ± 0.027	0.893 ± 0.017	0.994 ± 0.001	0.893 ± 0.017	0.974 ± 0.014	0.825 ± 0.027
	ESM2AbLang	0.962 ± 0.015	0.892 ± 0.029	0.880 ± 0.021	0.993 ± 0.001	0.878 ± 0.022	0.978 ± 0.013	0.798 ± 0.036
	ResPPI	0.866 ± 0.048	0.381 ± 0.094	0.323 ± 0.062	0.971 ± 0.002	0.244 ± 0.093	0.773 ± 0.105	0.152 ± 0.072
	PIPR	0.943 ± 0.018	0.808 ± 0.046	0.758 ± 0.049	0.987 ± 0.003	0.743 ± 0.058	0.959 ± 0.011	0.610 ± 0.077
	AbAgIntPre	0.968 ± 0.014	0.911 ± 0.029	0.904 ± 0.017	0.994 ± 0.001	0.906 ± 0.017	0.935 ± 0.024	**0.880 ± 0.034**
	MasonsCNN	0.964 ± 0.014	0.913 ± 0.017	0.892 ± 0.014	0.994 ± 0.000	0.895 ± 0.014	0.913 ± 0.035	0.879 ± 0.030
	DeepAAI*	—	—	—	—	—	—	—
	**DeepInterAware**	0.969 ± 0.015	**0.935 ± 0.029**	**0.924 ± 0.021**	**0.996 ± 0.001**	**0.925 ± 0.021**	0.978 ± 0.011	0.878 ± 0.040
SAbDab	DrugBAN	0.706 ± 0.020	0.676 ± 0.039	0.306 ± 0.034	0.649 ± 0.017	0.668 ± 0.016	0.621 ± 0.019	0.724 ± 0.027
	ESM2AntiBERTy	0.528 ± 0.005	0.512 ± 0.008	0.059 ± 0.011	0.529 ± 0.007	0.538 ± 0.038	0.516 ± 0.014	0.569 ± 0.098
	ESM2AbLang	0.629 ± 0.064	0.578 ± 0.063	0.204 ± 0.103	0.600 ± 0.053	0.617 ± 0.033	0.580 ± 0.052	0.662 ± 0.026
	ResPPI	0.582 ± 0.053	0.543 ± 0.053	0.132 ± 0.092	0.558 ± 0.051	0.616 ± 0.035	0.535 ± 0.048	0.734 ± 0.091
	PIPR	0.540 ± 0.011	0.518 ± 0.020	0.067 ± 0.016	0.531 ± 0.010	0.539 ± 0.033	0.519 ± 0.024	0.569 ± 0.086
	AbAgIntPre	0.778 ± 0.039	0.728 ± 0.062	0.413 ± 0.053	0.705 ± 0.028	0.711 ± 0.022	0.681 ± 0.038	0.746 ± 0.013
	MasonsCNN	0.697 ± 0.022	0.679 ± 0.026	0.314 ± 0.058	0.657 ± 0.029	0.630 ± 0.021	0.668 ± 0.039	0.598 ± 0.029
	DeepAAI	0.786 ± 0.021	0.754 ± 0.033	0.431 ± 0.032	0.714 ± 0.014	0.713 ± 0.017	0.692 ± 0.010	0.737 ± 0.038
	**DeepInterAware**	**0.827 ± 0.008**	**0.823 ± 0.019**	**0.483 ± 0.007**	**0.740 ± 0.004**	**0.744 ± 0.008**	**0.714 ± 0.011**	**0.776 ± 0.014**

The best results are marked in **bold** and the second‐best results are underlined. ‘‐’ represents that the method is unsuitable for the current task.

When antibodies bind to antigens, assessing the neutralization potential of antibodies is essential. The performances of all methods in neutralization prediction are demonstrated in **Table** **2**. Compared to the second‐best methods of each scenario, DeepInterAware achieves improvements of 1.4%, 3.7%, and 2.3% in AUROC, AUPRC, and MCC, respectively, under the Ab Unseen scenario; 1.8%, 1.3%, and 4.4% under the Ag Unseen scenario; 5.6%, 6.5%, and 9.9% under the Ag&Ab Unseen scenario. It can be observed that all methods perform better under the Ag unseen scenario than the Ab Unseen scenario, for the homology between antigens is higher than the homology between antibodies (Figure S1, Supporting Information). Notably, compared to the baseline methods, DeepInterAware performs particularly well in the Ag&Ab Unseen scenario, in which both the test antigens and antibodies are not included in the training set.

**Table 2:** Neutralization performance comparison on HIV dataset.

	Model	AUROC	AUPRC	MCC	ACC	F1	Precision	Recall
Ab Unseen	DrugBAN	0.784 ± 0.028	0.722 ± 0.044	0.402 ± 0.030	0.711 ± 0.024	0.606 ± 0.047	0.711 ± 0.111	0.555 ± 0.121
	ESM2AbLang	0.722 ± 0.001	0.617 ± 0.006	0.319 ± 0.001	0.669 ± 0.001	0.596 ± 0.002	0.602 ± 0.005	0.602 ± 0.005
	ESM2AntiBERTy	0.713 ± 0.022	0.611 ± 0.086	0.270 ± 0.030	0.642 ± 0.009	0.576 ± 0.055	0.549 ± 0.063	0.613 ± 0.083
	ResPPI	0.724 ± 0.030	0.655 ± 0.064	0.296 ± 0.071	0.661 ± 0.029	0.525 ± 0.196	0.629 ± 0.076	0.542 ± 0.294
	PIPR	0.722 ± 0.043	0.654 ± 0.052	0.309 ± 0.070	0.675 ± 0.050	0.514 ± 0.069	0.675 ± 0.056	0.424 ± 0.100
	AbAgIntPre	0.725 ± 0.009	0.661 ± 0.063	0.281 ± 0.057	0.659 ± 0.037	0.353 ± 0.099	**0.787 ± 0.079**	0.233 ± 0.084
	MasonsCNN	0.793 ± 0.036	0.721 ± 0.043	0.443 ± 0.061	0.732 ± 0.031	0.667 ± 0.046	0.680 ± 0.064	0.659 ± 0.054
	DeepAAI	0.812 ± 0.030	0.741 ± 0.033	0.470 ± 0.049	0.741 ± 0.026	0.693 ± 0.040	0.677 ± 0.062	0.712 ± 0.044
	**DeepInterAware**	**0.826 ± 0.017**	**0.778 ± 0.018**	**0.493 ± 0.040**	**0.753 ± 0.023**	**0.713 ± 0.030**	0.710 ± 0.044	**0.716 ± 0.031**
Ag Unseen	DrugBAN	0.842 ± 0.006	0.816 ± 0.008	0.496 ± 0.009	0.744 ± 0.007	0.683 ± 0.027	**0.812 ± 0.026**	0.592 ± 0.057
	ESM2AbLang	0.840 ± 0.001	0.815 ± 0.002	0.515 ± 0.005	0.758 ± 0.003	0.745 ± 0.011	0.728 ± 0.012	0.763 ± 0.001
	ESM2AntiBERTy	0.842 ± 0.009	0.817 ± 0.011	0.517 ± 0.019	0.757 ± 0.009	0.750 ± 0.010	0.719 ± 0.013	**0.784 ± 0.016**
	ResPPI	0.796 ± 0.071	0.768 ± 0.076	0.415 ± 0.144	0.701 ± 0.079	0.566 ± 0.211	0.796 ± 0.050	0.486 ± 0.255
	PIPR	0.839 ± 0.012	0.816 ± 0.012	0.503 ± 0.026	0.751 ± 0.015	0.720 ± 0.030	0.757 ± 0.051	0.695 ± 0.083
	AbAgIntPre	0.855 ± 0.010	0.837 ± 0.008	0.543 ± 0.013	0.772 ± 0.007	0.730 ± 0.008	0.810 ± 0.019	0.665 ± 0.023
	MasonsCNN	0.839 ± 0.006	0.813 ± 0.008	0.516 ± 0.010	0.759 ± 0.005	0.740 ± 0.009	0.741 ± 0.011	0.740 ± 0.025
	DeepAAI	0.829 ± 0.010	0.799 ± 0.013	0.498 ± 0.014	0.750 ± 0.008	0.733 ± 0.007	0.726 ± 0.019	0.742 ± 0.030
	**DeepInterAware**	**0.873 ± 0.008**	**0.850 ± 0.012**	**0.587 ± 0.019**	**0.794 ± 0.010**	**0.778 ± 0.014**	0.779 ± 0.019	0.778 ± 0.041
Ag&Ab Unseen	DrugBAN	0.691 ± 0.039	0.700 ± 0.057	0.274 ± 0.062	0.614 ± 0.042	0.500 ± 0.054	0.735 ± 0.104	0.394 ± 0.102
	ESM2AbLang	0.675 ± 0.003	0.678 ± 0.004	0.266 ± 0.005	0.632 ± 0.001	0.604 ± 0.003	0.652 ± 0.002	0.565 ± 0.005
	ESM2AntiBERTy	0.657 ± 0.062	0.657 ± 0.079	0.247 ± 0.087	0.620 ± 0.047	0.580 ± 0.078	0.647 ± 0.061	0.532 ± 0.104
	ResPPI	0.647 ± 0.025	0.619 ± 0.046	0.184 ± 0.041	0.590 ± 0.027	0.432 ± 0.095	0.648 ± 0.037	0.332 ± 0.105
	PIPR	0.681 ± 0.068	0.699 ± 0.065	0.280 ± 0.086	0.632 ± 0.050	0.573 ± 0.034	0.694 ± 0.067	0.492 ± 0.047
	AbAgIntPre	0.678 ± 0.062	0.703 ± 0.071	0.264 ± 0.098	0.596 ± 0.062	0.379 ± 0.137	**0.805 ± 0.084**	0.257 ± 0.111
	MasonsCNN	0.696 ± 0.037	0.694 ± 0.063	0.274 ± 0.071	0.630 ± 0.034	0.595 ± 0.058	0.662 ± 0.077	0.551 ± 0.102
	DeepAAI	0.668 ± 0.062	0.680 ± 0.069	0.249 ± 0.100	0.609 ± 0.055	0.520 ± 0.094	0.683 ± 0.096	0.436 ± 0.130
	**DeepInterAware**	**0.752 ± 0.040**	**0.768 ± 0.049**	**0.379 ± 0.066**	**0.684 ± 0.039**	**0.646 ± 0.074**	0.735 ± 0.061	**0.590 ± 0.118**

The best results are marked in **bold** and the second‐best results are underlined.

In practical applications, a desirable method should have good transferability for novel antigens. Here, we explore the effectiveness of leveraging knowledge acquired from the HIV dataset to enable predictions of antibody neutralization capabilities against the SARS‐CoV‐2 virus (included in the CoV‐AbDab dataset). Despite the distinct infection mechanisms between HIV and SARS‐CoV‐2, both viruses are categorized under the RNA virus class, suggesting that insights gained from HIV could inform predictions regarding SARS‐CoV‐2 neutralization. The performances of all models' transferability are demonstrated in Table S1 (Supporting Information). DeepInterAware, alongside other baseline methods, undergoes initial training using the HIV dataset to create pre‐trained models, and these models are then further fine‐tuned on the CoV‐AbDab training set and subsequently assessed on the CoV‐AbDab test set. Compared to the second‐best methods, DeepInterAware shows improvements of 0.9% and 2.7% in AUPRC and MCC, respectively, and achieves a competitive result in AUROC scores (0.838), demonstrating DeepInterAware's exceptional transferability, enabling it to be effectively adapted to new antigens. This feature is particularly advantageous in real‐world applications, where new antigens have only a few known binding or neutralization antibodies.

As shown in Figure 1, DeepInterAware contains several well‐designed modules, such as IIL, SIL, and DCF, which contribute to its superior performance. The usefulness of these critical modules is evaluated in the ablation study (Section S3.5, Supporting Information).

### 2.3 DeepInterAware Identifies Potential Binding Sites of Antigen–Antibody Interactions

Understanding the underlying mechanisms of AAIs is crucial for accurately predicting them. Most existing prediction methods solely focus on determining whether an interaction will occur without providing insights into the binding sites. DeepInterAware introduces a well‐designed IIL, which employs a bilinear attention mechanism to identify the amino acids within Ag‐Ab sequences that play critical roles in these interactions by assigning weights to every amino acid. It helps uncover potential binding sites (epitopes and paratopes) of antigens and antibodies, thereby enhancing the accuracy of predictions.

**Figure 2:**
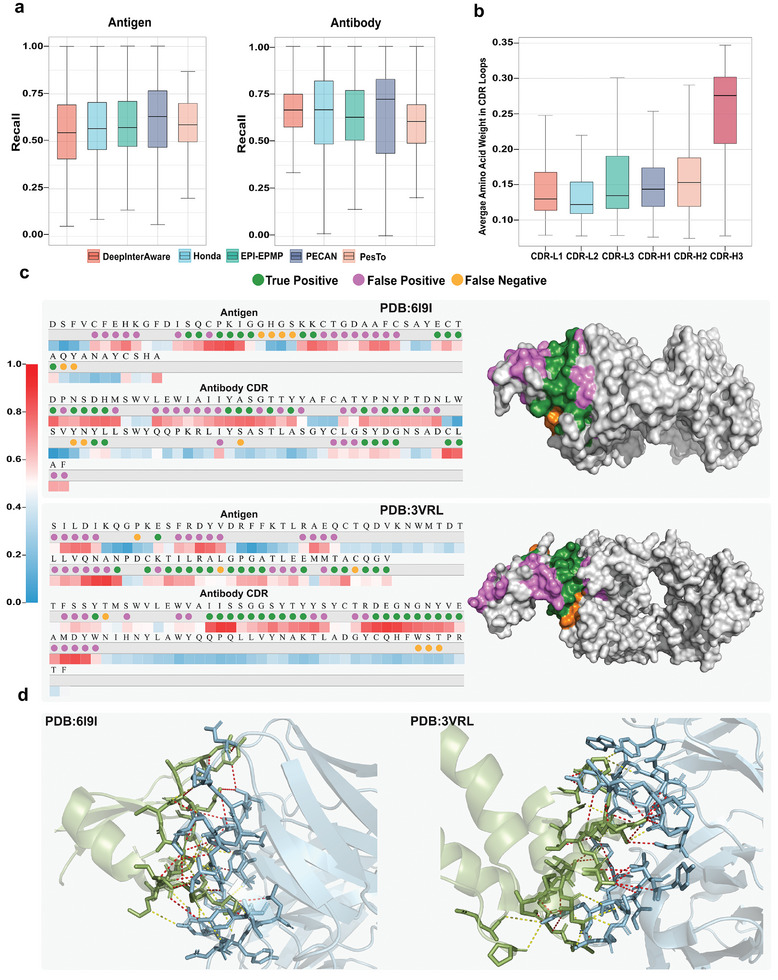
DeepInterAware captures the underlying knowledge of AAIs. a) The recall rate distribution of binding sites identified by different methods. b) The average amino acid weights in different CDR loops of antibodies. c) The visualization of the identified binding sites on the antigen sequences, antibody CDR sequences, and binding interfaces of two complexes (PDB:6I9I and PDB:3VRL). The correctly predicted binding sites (True Positive) are highlighted in green; the amino acids wrongly predicted as the binding sites (False Positive) are highlighted in pink; the binding sites wrongly predicted as the non‐binding (False Negative) are highlighted in orange. d) The visualization of predicted epitope‐paratope binding pairs in these two complexes. The antibodies and antigens are highlighted in blue and green, respectively. The red dashed lines indicate correctly predicted epitope‐paratope binding pairs, while the yellow dashed lines are missed binding pairs.

To evaluate the performance of DeepInterAware in binding site identification, we used the model trained on Ag–Ab sequences of the SAbDab dataset and employed the IIL to obtain the weights of amino acids and converted these weights into values ranging from 0 to 1, which represent the probabilities of being binding sites (see Supporting Information, Section S3.2 for more details). The SAbDab dataset includes the Ag–Ab complex structures, offering real binding sites to validate the model's performance. For comparison, we considered the state‐of‐the‐art binding site prediction methods, including the sequence‐based method (Honda et al.'s method^[^ ^26^ ^]^) as well as the structure‐based methods EPI‐EPMP,^[^ ^27^ ^]^ PECAN,^[^ ^28^ ^]^ and additionally adopted the structure‐based protein binding site prediction method PesTo.^[^ ^29^ ^]^ For DeepInterAware, residues with a probability greater than 0.5 are considered as binding sites, while thresholds for other methods are determined according to their publications or publicly available source codes. **Figure** **2**a illustrates the recall rate distribution for binding sites predicted by different methods, and the results show that our method could find out most real binding sites, with recall scores comparable to those of other methods. Additionally, we considered a variant of DeepInterAware named DeepInterAware*, which utilizes the annotated binding sites for model training, as the compared methods do, and the AUROC and AUPRC scores of all methods are reported in Table S2 (Supporting Information). Overall, all methods produce better results in paratope prediction than epitope prediction, likely due to the longer lengths of antigens. Although DeepInterAware performs much poorer than the binding site prediction methods, its variant DeepInterAware* can deliver the best or second‐best results among all methods, demonstrating that DeepInterAware can be extended to achieve even greater prediction accuracy when annotated binding sites are used during model training.

Numerous studies have shown that the CDR loops in the heavy chain are more frequently involved in the AAIs compared to those in the light chain,^[^ ^30^, ^31^, ^32^ ^]^ and CDR‐H3, positioned at the core of the antigen binding site, directly influences the antigen‐binding propensity of all CDR loops due to its length and conformational diversity.^[^ ^30^ ^]^ To investigate whether DeepInterAware could focus on the critical loops, we obtained the amino acid weights of SAbDAb dataset's antibody sequences learned by DeepInterAware, and conducted a detailed analysis of the average weights of amino acids from different loops of antibodies. As shown in Figure 2b, the amino acids on CDR‐H3 have the greatest average weights, and the amino acids on CDR‐H2 have the second‐greatest. In contrast, the amino acids on the light chain, especially CDR‐L2, have the lowest average weights among the six loops. This finding is consistent with existing studies^[^ ^30^, ^31^, ^32^, ^33^ ^]^ based on binding energy analysis, which reveals that CDR‐H3 and CDR‐H2 contribute the most to the Ag–Ab binding free energy, while CDR‐L2 contributes the least.

To show the identified binding sites more intuitively, we utilize two Ag‐Ab complexes: Rift Valley fever virus (PDB: 6I9I) and BMJ4 p24 capsid protein (PDB: 3VRL) for analysis. Focusing on amino acids with probabilities exceeding 0.5 as potential binding sites, Figure 2c shows the visualization of the identified binding sites on the antigen sequences, antibody CDR sequences, and binding interfaces of two complexes: 6I9I and 3VRL. In the case of the complex 6I9I, our DeepInterAware successfully identified 12 out of 18 epitopes and an impressive 23 out of 26 paratopes. For the complex 3VRL, DeepInterAware accurately predicted 18 out of 21 epitopes, and effectively recognized 21 out of 24 paratopes. It is noteworthy that amino acids in close proximity to these binding sites exhibit significant attention scores, indicating that while they may not be directly involved in AAIs, they could play a pivotal role through mechanisms such as long‐range electrostatic effects and surface‐solvent interactions, as suggested by the studies.^[^ ^34^, ^35^, ^36^ ^]^ Building upon the identification of these binding sites, we used PyMOL to visualize the predicted epitope‐paratope binding pairs for the complexes: 6I9I and 3VRL, as shown in Figure 2d. DeepInterAware successfully predicted 24 epitope‐paratope binding pairs (recall rate of 0.56) for complex 6I9I with 43 experimentally validated pairs and 29 pairs (recall rate of 0.64) for complex 3VRL with 45 validated pairs.

In summary, DeepInterAware can effectively capture the underlying mechanism of AAIs, even in the absence of explicit structure data, and deliver useful insights for the prediction of these interactions.

### 2.4 DeepInterAware Detects Mutations Within Antigens or Antibodies and Predicts the Binding Free Energy Changes

The amino acid mutations of antigens and antibodies can significantly alter AAIs. DeepInterAware is capable of detecting mutations within antigens or antibodies, and can be extended to predict the binding free energy changes upon these mutations.

**Figure 3:**
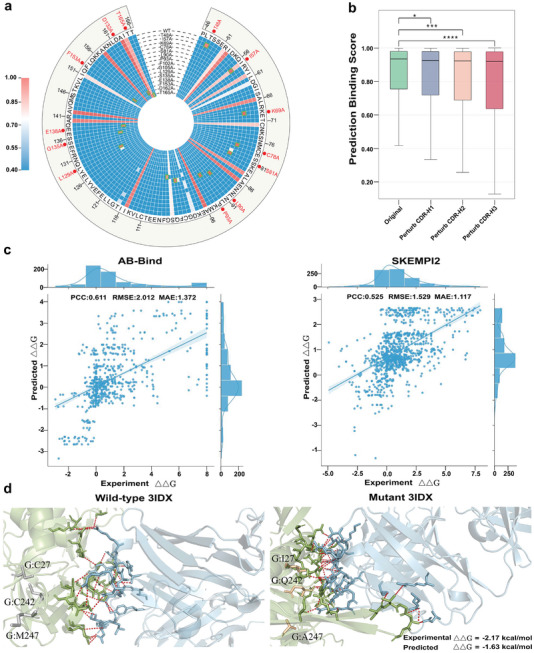
DeepInterAware detects the mutations on antigen and antibody sequences. a) The circular heatmap illustrates amino acid attention scores in mutation regions, with the outer ring representing the wild‐type (WT) and the inner rings representing 15 mutants, each containing a single amino acid substitution. The green box indicates the mutated amino acid spotted by DeepInterAware, while the red dot pinpoints the mutation location. b) The distribution of predicted binding scores for perturbed antibodies under perturbations in three loops, with significance indicated by *, ***, and **** for *P*‐values ⩽ 0.05, ⩽ 0.001, and ⩽ 0.0001, respectively. c) The regression correlation between predicted and experimental values of changes in binding free energy on the AB‐Bind and SKEMPI2 datasets. d) An example of experimentally determined and predicted binding free energy changes of the wild‐type of the Ag–Ab complex (PDB:3IDX) and its mutant, respectively, where the gray and orange sticks represent residues before and after mutating, the green sticks represent epitopes, the blue sticks represent paratopes, and the red dashed lines indicate the interactions of epitope‐paratope binding pairs.

The mutation within antigens can substantially compromise the efficacy of existing antibody therapies and vaccines. Here, we used the AVIDa‐hIL6 test set, which contains the wild‐type (WT) antigen and its mutants with single amino acid mutations within the 46–116 amino acid range, to test the DeepInterAware's capacity of detecting antigen mutations. **Figure** **3**a shows a circular heatmap of the amino acid attention scores in the mutation regions of the WT antigen and its mutants. Compared to the WT antigen, its mutants exhibit similar attention scores for most amino acids due to the high sequence homology among them, but the attention scores of the mutation amino acids are significantly greater than those of the WT, and the significant differences between WT and mutants could help to detect mutations within the antigen.

The antibody CDR loops, especially CDR‐H3, play a pivotal role in specificity recognition, and the mutations in CDR loops of the binding antibodies may likely lead to non‐binding antibodies. Here, we used the AVIDa‐hIL6 test set and perturbed the CDR loops of binding antibodies to simulate dysfunctional antibodies. Specifically, we generated three mutants for each binding antibody by randomly mutating one amino acid within each of the three CDR loops of the heavy chain, and then we used these perturbed antibodies to test DeepInterAware's capacity of detecting antibody mutations. We employed DeepInterAware to predict the binding scores of perturbed antibodies, which reflect the probability of Ag–Ab binding, and Figure 3b illustrates the distribution of predicted binding scores for perturbed antibodies under perturbations in three loops. The results show that perturbed antibodies generally have lower binding scores than original antibodies, especially mutations in the CDR‐H3 loop (*p*‐value ⩽ 0.0001) exhibit significant performance differences. The results demonstrate that our method is sensitive to perturbations in CDR loops and effectively captures differences caused by antibody mutations.

Mutations within antigens or antibodies can alter the conformation of Ag–Ab complexes^[^ ^37^ ^]^ and affect their binding free energy, highlighting the importance of accurately predicting these changes. As shown in Figure S2 (Supporting Information), we extended DeepInterAware's architecture to predict changes in binding free energy and evaluated the extended model using the AB‐Bind and SKEMPI datasets (ΔΔ*G*, *kcal* · *mol* ^−1^). Figure 3c compares the predicted and experimentally measured binding free energy changes, and our extended model achieved 0.611, 2.012 *kcal* · *mol* ^−1^, and 1.372 *kcal* · *mol* ^−1^ in PCC, RMSE, and MAE on the AB‐Bind dataset, 0.525, 1.529 *kcal* · *mol* ^−1^, and 1.117 *kcal* · *mol* ^−1^ in PCC, RMSE, and MAE on the SKEMPI2 dataset. An example of the Ag‐Ab complex (PDB: 3IDX) and its mutant analyzed by our method is shown in Figure 3d. As shown in Table S3 (Supporting Information), we compared extended DeepInterAware with some state‐of‐the‐art binding free energy change prediction methods, including FoldX,^[^ ^38^ ^]^ EvoEF,^[^ ^39^ ^]^ and AttABseq,^[^ ^40^ ^]^ and the results show that our method outperforms all compared methods in terms of three metrics, when evaluated by 10‐fold cross‐validation on both datasets.

The above results and discussions provide a deeper understanding of DeepInterAware's capabilities in detecting mutations within antigens or antibodies. Furthermore, we analyzed its scalability in predicting binding free energy changes upon these mutations. These strengths are especially valuable for advancing antibody affinity maturation and promoting the development of therapeutic antibodies.

### 2.5 DeepInterAware Screens the Potential Antibodies Binding to HER2 Target

Screening candidate antibodies for antigen binding is critical in antibody‐drug development. HER2 (human epidermal growth factor receptor 2), a pivotal protein associated with tumor development and progression via mutation or overexpression, is a key target for therapies in cancers such as breast and gastric. The rise of drug resistance to existing HER2‐targeted treatments underscores the urgent need for innovative therapeutic strategies to satisfy significant market needs. Here, we demonstrate how to utilize our developed method to screen antibodies binding to the HER2 target.

**Figure 4:**
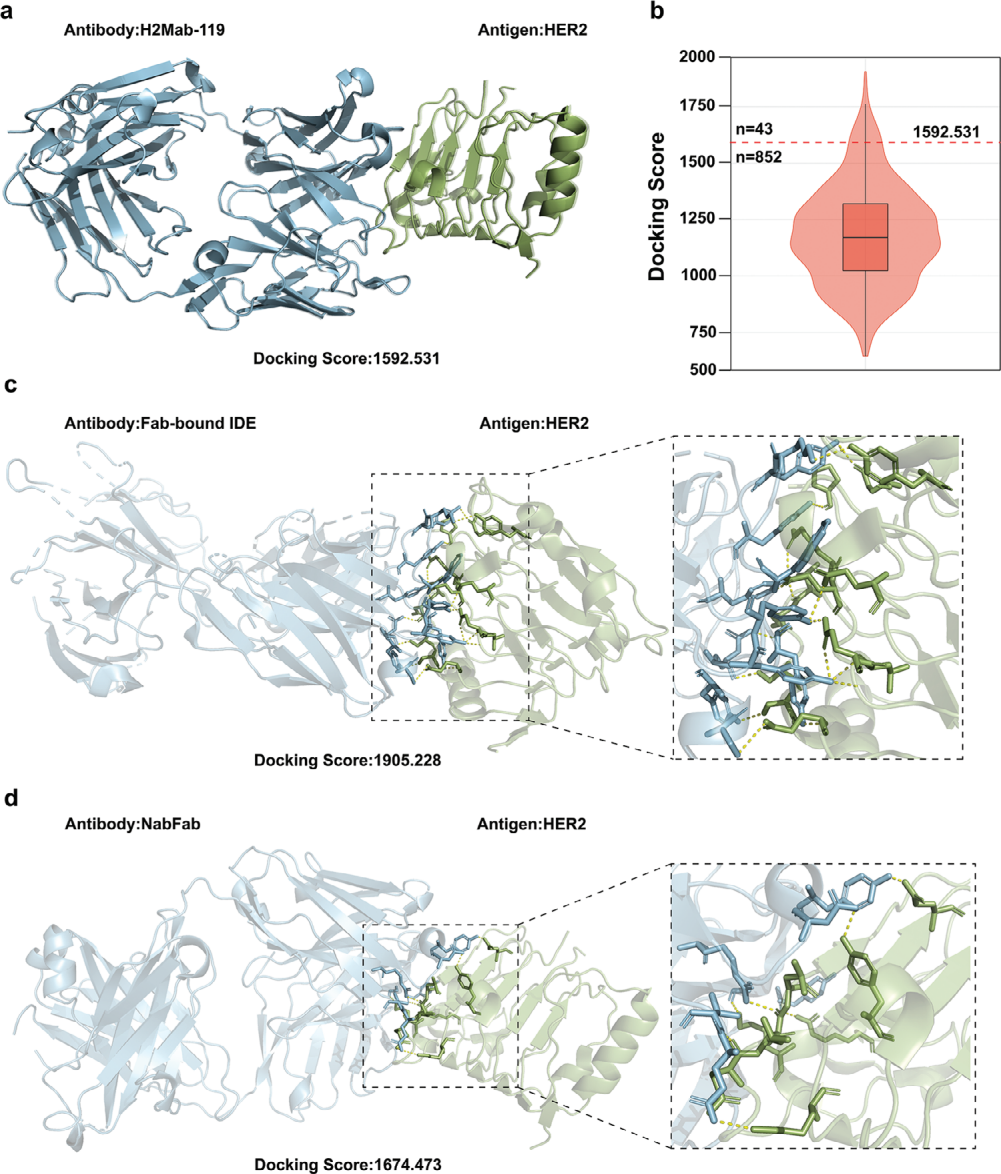
Discovery of potential antibodies binding to the target HER2. a) The docking pose of an authentic complex (PDB: 8JYR) composed of antigen HER2 and antibody H2Mab‐119. b) The docking score distribution of HER2 and all candidate antibodies with high binding scores. c) The docking pose of the antibody Fab‐bound IDE and HER2 obtained the highest docking score among all candidate antibodies. d) The docking pose of the antibody NabFab and HER2, whose binding region is similar to the region that HER2 binds to the cancer‐specific therapeutic antibody H2Mab‐119.

H2Mab‐119 is a cancer‐specific therapeutic antibody that targets HER2, and their complex structure (PDB: 8JYR) is available in the SAbDab database. As shown in **Figure** **4**a, we decomposed the complex structure into antigen and antibody components, and employed the ZDOCK to calculate the global docking score, which served as our evaluation benchmark. Then, we used DeepInterAware trained on the SAbDab dataset, to predict the binding scores of HER2 with 11 683 antibodies from the SAbDab database. A total of 895 antibodies with binding scores greater than 0.95 were considered as candidates, which were subsequently global docked with HER2 using ZDOCK. It is worth noting that the HER2 antigen and its binding antibodies were excluded from the SAbDab dataset during model training. Figure 4b illustrates the distribution of docking scores of 895 complexes with screened antibodies and HER2, and 43 complexes among them achieved docking scores higher than that of the complex 8YJR, and details about these complexes and their docking scores are provided in Section S3.4 (Supporting Information). Figure 4c shows the docking pose of the complex with the highest docking score, identifying Fab‐bound IDE (PDB: 4M1C) as its antibody, with their interaction interface highlighted. Additionally, we found an interesting phenomenon shown in Figure 4d: the region on HER2 that binds with the antibody NabFab (PDB: 7RTH) is similar to the region it binds to the cancer‐specific therapeutic antibody H2Mab‐119, yet with a higher binding score. Figure S3 (Supporting Information) illustrates the alignment of the two regions, which exhibits 85% amino acid residue overlap, indicating that NabFab could be a new candidate antibody with potential efficacy in targeting HER2 for cancer therapy.

## 3 Conclusion

Predicting antigen–antibody interactions (AAIs) is crucial for advancing human therapeutics. While structure data is limited, abundant sequence data provides opportunities to develop computational methods. In the absence of structure data, fully leveraging sequence information to predict AAIs remains a promising research direction. In this study, we introduced DeepInterAware, a method designed to accurately predict AAIs using only sequence data. Despite relying solely on sequence information, DeepInterAware effectively captures structural insights, enhancing AAI prediction accuracy and offering additional advantages. These include uncovering the underlying mechanisms of AAIs, identifying potential binding sites, and detecting mutations in antigens or antibodies. Moreover, DeepInterAware can be extended to predict changes in binding free energy resulting from mutations. We employed DeepInterAware to screen antibodies for HER2, a key target for cancer therapies, and discovered a new candidate antibody, NabFab, which shows potential efficacy in targeting HER2 for cancer treatment. These strengths make DeepInterAware a powerful tool with significant potential for advancing therapeutic antibody development.

## 4 Experimental Section

### Problem Formulation

In a typical AAI scenario, a set G of antigen sequences was considered, a set B of antibody sequences, an AAI matrix $Y\in {\left(0,1\right)}^{\left|G|\times |B\right|}$, where |G| and |B| denotes the number of antigens and antibodies. In the matrix $Y,Y_{\left(i,j\right)}=1$, if the i‐th antigen interacts with the j‐th antibody.

Given an antigen sequence $x_{Ag}\in G$ and an antibody sequence $x_{Ab}\in B$, The objective is to learn a mapping function $𝛩\left(\omega \right):E\left(x_{Ag},x_{Ab}\right)\to p$ that transforms an Ag–Ab pair into an interaction probability score p∈[0,1].

### DeepInterAware Architecture

As illustrated in Figure 1a, the proposed DeepInterAware contains four major modules: 1) Sequence Encoder; 2) Interaction Interface‐aware Learner (IIL); 3) Specificity Information Learner (SIL); 4) Dynamic Confidence Fusion (DCF) and Prediction. They are briefly introduced as follows.

### DeepInterAware Architecture—Sequence Encoder

Initially, given an Ag–Ab pair $\left\{x_{\mathrm{Ag}},x_{\mathrm{Ab}}\right\}$ with the true label $y^{*}$, advantage of protein language model ESM‐2 was taken to derive the sequence embedding matrix for $x_{\mathrm{Ag}}$, denoted as $X_{Ag}\in R^{M\times 480}$, which captures the essential characteristics of the antigens in a numerical format. Concurrently, the specialized antibody language model AbLang was used to generate the sequence embedding matrix for $x_{\mathrm{Ab}}$, represented as $X_{Ab}\in R^{N\times 768}$. Each row in these matrices corresponds to an amino acid feature, encapsulating its distinctive features within a vectorized form. Subsequently, a CNN with the architecture depicted in Figure S2c (Supporting Information) was utilized with the above embeddings as input, resulting in representations,

(1) $$\begin{array}{cc} H_{Ag} & ={\mathrm{CNN}}_{Ag}\left(X_{Ag}\right),\\ H_{Ab} & ={\mathrm{CNN}}_{Ab}\left(X_{Ab}\right) \end{array}$$

where $H_{Ag}\in R^{M\times d}$ and $H_{Ab}\in R^{N\times d}$ are representations of the amino acids in each antigen and antibody sequence, and M and N denote the number of amino acids.

### DeepInterAware Architecture—Interaction Interface‐Aware Learner

A bilinear attention network was applied to build the IIL, which captures the interaction interface information and pairwise local interactions between antigens and antibodies. The IIL consists of two layers: a bilinear interaction map to capture pairwise attention weights and an interaction information pooling module over the interaction information to sequence‐level representations.

The antigen and antibody sequence encoders generate the hidden representations $H_{\mathrm{Ag}}$ and $H_{\mathrm{Ab}}$ for every antigen and antibody sequence, and the bilinear interaction map was constructed using these hidden representations to obtain pairwise interaction matrix $I\in R^{M\times N}$,

(2) $$I=\left(\left(1\cdot q^{⊤}\right)\circ \sigma \left(H_{Ag}U\right)\right)\cdot \sigma \left(VH_{Ab}^{⊤}\right)$$

where $U\in R^{d\times K}\mathrm{and}V\in R^{K\times d}$ are learnable weight matrices for antigen and antibody representations, $q\in R^{K}$ is a learnable weight vector, 1 is a fixed all‐ones vector, ∘ denotes Hadamard (element‐wise) product. The elements in I indicate the interaction intensity of respective Ag–Ab residue pairs, with mapping to potential binding sites.

Based on the interaction map I, the antigen and antibody features were projected into the other feature space, and the features $H_{Ag}^{I}$ and $H_{Ab}^{I}$ were obtained, which contains AAI information and represents the contribution of each amino acid in the sequence to the interaction prediction, and both matrices have the same dimensions as $H_{\mathrm{Ag}}$ and $H_{\mathrm{Ab}}$, respectively,

(3) $$\begin{array}{cc} H_{Ag}^{I} & =I\cdot H_{Ab},\\ H_{Ab}^{I} & =I^{⊤}\cdot H_{Ag} \end{array}$$

We then obtain sequence‐level features $S_{Ab-Ag}^{I}\in R^{1\times d}$ and $S_{Ag-Ab}^{I}\in R^{1\times d}$ using Interaction Information Pooling (IIP) (Figure S2a, Supporting Information), by Equation (4). In view of this, we are able to display the weight of each amino acid calculated, thereby facilitating the learning of the interaction map I and improving the awareness of the interaction interface,

(4) $$\begin{array}{cc} S_{Ab-Ag}^{I} & =\mathrm{Softmax}\left(\phi \left(H_{Ag}^{I}\right)\right)\cdot H_{Ag},\\ S_{Ag-Ab}^{I} & =\mathrm{Softmax}\left(\phi \left(H_{Ab}^{I}\right)\right)\cdot H_{Ab} \end{array}$$

where ϕ(·) is the Activation Unit with the architecture in Figure S2d (Supporting Information) to calculate the weights of amino acids based on interaction feature $H_{Ag}^{I}$ and $H_{Ab}^{I}$.

The final interaction information feature of Ag‐Ab pair $Z^{I}\in R^{1\times D}$ can be obtained according to Equations (5),

(5) $$\begin{array}{cc} S^{I} & =S_{Ab-Ag}^{I}∥S_{Ag-Ab}^{I},\\ Z^{I} & =\rho^{I}\left(S^{I}\right) \end{array}$$

where the $\rho^{I}\left(\cdot \right)$ represents an MLP, which is used to convert the concatenation features of antigen and antibody into pair features $Z^{I}$ and its architecture can be viewed in Figure S2e (Supporting Information). D is the feature dimension after projection.

### DeepInterAware Architecture—Specificity Information Learner

The SIL directly calculates the sequence‐level features $S_{Ag}^{S}$ and $S_{Ab}^{S}$ based on the inherent features $H_{\mathrm{Ag}}$, $H_{\mathrm{Ab}}$ of antigens and antibodies by Equation (6),

(6) $$\begin{array}{cc} S_{Ag}^{S} & =\mathrm{Softmax}\left(\phi \left(H_{Ag}\right)\right)\cdot H_{Ag},\\ S_{Ab}^{S} & =\mathrm{Softmax}\left(\phi \left(H_{Ab}\right)\right)\cdot H_{Ab} \end{array}$$

Similar to Equations (5), specificity features $Z^{S}$ can be obtained. The essence of AA‐wise Self‐attention Pooling (ASP) (Figure S2b, Supporting Information), lies in its ability to adaptively adjust weights according to the characteristics of the data, thus more accurately reflecting the actual contribution of amino acids in AAIs.

### DeepInterAware Architecture—Dynamic Confidence Fusion and Prediction

In this section, we elaborate on how to conduct dynamic confidence fusion based on the feature and feature confidence. Inspired by Han et al.'s^[^ ^41^ ^]^ research, we utilize True Class Probability (TCP)^[^ ^42^ ^]^ to quantify the feature confidence. Based on the interaction features $Z^{I}$ and specificity features $Z^{S}$, we use two three‐layer MLPs $f^{I}\left(\cdot \right)$  and $f^{S}\left(\cdot \right)$  as classifiers to predict the Ag–Ab binding probabilities $p^{I}$ and $p^{S}$,

(7) $$\begin{array}{c} p^{I}=f^{I}\left(Z^{I}\right),\\ p^{S}=f^{S}\left(Z^{S}\right) \end{array}$$

Here, we take the prediction of the classifier $f^{I}\left(\cdot \right)$  as the example. For an Ag‐Ab pair, the TCP score c is p if the pair is binding (i.e., $y^{*}=1$), and 1−p otherwise (i.e., $y^{*}=0$), which is formally described as Equations (8). When the classifier misclassifies a sample, the probability associated with the true class can be close to a lower value, thereby reflecting the classifier's error.

(8) $$c^{I}=\left\{\begin{array}{cc} p^{I} & \mathrm{if}\;y^{*}=1\\ 1-p^{I} & \mathrm{if}\;y^{*}=0 \end{array}\right.$$

Similarly, we calculate the TCP score $c^{S}$ of the prediction $p^{S}$ yielded by the classifier $f^{S}\left(\cdot \right)$ .

Although TCP offers enhanced reliability in confidence estimation, its application during testing is precluded by the need for labeled data. Thus, we employ two neural networks $g^{I}\left(\cdot \right)$ and $g^{S}\left(\cdot \right)$ with one fully connected layer followed by a sigmoid function to predict TCP scores of predictions from $Z^{I}$ and $Z^{S}$,

(9) $$\begin{array}{cc} {\hat{c}}^{I} & =g^{I}\left(Z^{I}\right),\\ {\hat{c}}^{S} & =g^{S}\left(Z^{S}\right) \end{array}$$

where ${\hat{c}}^{I}$ and ${\hat{c}}^{S}$ are the predicted TCP scores, which should be approximated to $c^{I}$ and $c^{S}$.

Finally, the dynamic fused features are obtained based on the interaction features, specificity features and their confidence scores, and the ultimate interaction probability p is subsequently determined using the three‐layer MLP classifier f(·),

(10) $$\begin{array}{cc} p & =f({\hat{c}}^{I}⊗Z^{I}∥{\hat{c}}^{S}⊗Z^{S}) \end{array}$$

### DeepInterAware Architecture—Model Training

For training the classifiers $f^{I}\left(\cdot \right)$  and $f^{S}\left(\cdot \right)$ , we consider the Binary CrossEntropy Loss (BCE) loss functions for the predicted probabilities $p^{I}$ and $p^{S}$ and real labels $y^{*}$ of training pairs,

(11) $$\begin{array}{cc} L_{I}^{cls} & =BCE(y^{*},p^{I}),\\ L_{S}^{cls} & =BCE(y^{*},p^{S}) \end{array}$$

For training the TCP predictors $g^{I}\left(\cdot \right)$ and $g^{S}\left(\cdot \right)$, we use the Mean Square Error (MSE) loss function,

(12) $$\begin{array}{cc} L_{I}^{conf} & =MSE({\hat{c}}^{I},c^{I}),\\ L_{S}^{conf} & =MSE({\hat{c}}^{S},c^{S}) \end{array}$$

For training the fused feature‐based classifier f(·), we use the BCE loss function,

(13) $$\begin{array}{cc} L_{Fusion}^{cls} & =BCE(y^{*},p) \end{array}$$

The overall loss can be formulated as follows:

(14) $$\begin{array}{cc} L=\left(L_{I}^{cls}+L_{S}^{cls}\right)*\lambda_{1} & +\left(L_{I}^{conf}+L_{S}^{conf}\right)*\lambda_{2}+L_{Fusion}^{cls}*\lambda_{3} \end{array}$$

where $\lambda_{1}$, $\lambda_{2}$ and $\lambda_{3}$ are hyperparameters used to balance different losses.

The model training consists of two stages. First, the IIL and SIL modules are trained separately using BCE loss in Equation (11). Then, the entire model is trained with the loss function defined in Equation (14).

### Experimental Setting—Datasets

In this study, several datasets were used to evaluate DeepInterAware's performances and support these findings. Specifically, the AVIDa‐hIL6^[^ ^43^ ^]^ and SAbDab^[^ ^44^ ^]^ datasets were employed for binding prediction, HIV^[^ ^45^ ^]^ and CoV‐AbDab^[^ ^46^ ^]^ datasets for neutralization prediction. Additionally, the SAbDab dataset was adapted for binding site prediction, AB‐Bind^[^ ^47^ ^]^ and SKEMPI2^[^ ^48^ ^]^ datasets for binding free energy change prediction.

### Experimental Setting—Baseline Methods

For the AAI prediction, DeepInterAware was compared with several state‐of‐the‐art methods. These include AAI prediction methods such as DeepAAI,^[^ ^9^ ^]^ AbAgIntPre,^[^ ^8^ ^]^ and MasonCNN.^[^ ^6^ ^]^ Additionally, several biomolecular interaction prediction methods were considered, such as protein–protein interaction prediction methods like PIPR^[^ ^49^ ^]^ and ResPPI,^[^ ^50^ ^]^ as well as the drug‐target interaction prediction method DrugBAN,^[^ ^51^ ^]^ and also used pre‐trained language models ESM2,^[^ ^10^ ^]^ AbLang^[^ ^21^ ^]^ and AntiBERTy^[^ ^22^ ^]^ to build baselines ESM2AbLang and ESM2AntiBERTy. For the binding site identification, the state‐of‐the‐art binding site prediction methods were considered, including the sequence‐based method (Honda et al.'s method^[^ ^26^ ^]^) as well as the structure‐based methods EPI‐EPMP,^[^ ^27^ ^]^ PECAN,^[^ ^28^ ^]^ and PesTo.^[^ ^29^ ^]^ For the binding free energy change prediction, several state‐of‐the‐art binding free energy change prediction methods were considered, including the force field‐based methods FoldX^[^ ^38^ ^]^ and EvoEF,^[^ ^39^ ^]^ and deep learning‐based method AttABseq.^[^ ^40^ ^]^

### Experimental Setting—Model Evaluation Metrics

To ensure the effectiveness of performance comparison, five independent experiments were conducted with distinct random seeds for both the binding prediction and neutralization prediction, fivefold cross‐validation for the binding site identification and tenfold cross‐validation for the binding free energy change prediction. The reported results include the average and variance of these experimental outcomes. The AUROC (Area Under the Receiver Operating characteristic Curve), AUPRC (Area Under the Precision‐call Curve), and MCC (Matthews Correlation Coefficient) are used as the primary metrics to evaluate the model's performance on binding and neutralization tasks. The AUROC and AUPRC are used as the primary metrics to evaluate the model's performance on binding site prediction task. The PCC (Pearson Correlation Coefficient), RMSE (Root‐Mean‐Square Error), and MAE (Mean Absolute Error) are used as the primary metrics to evaluate the model's performance on binding free energy change prediction task.

More details about dataset collection and splitting, baseline methods and their implementation, hyperparameters settings can be found in Section S1 (Supporting Information).

## Conflict of Interest

W.Z. and Y.X. are inventors on patent applications related to this work filed by Wuhan Huamei Biotech Co., Ltd. (Chinese patent application nos. 2023.12.21 202311783760.X). The authors declare no other competing interests.

## Author Contributions

Y.X. and Z.W. contributed equally to this work. Y.X. designed and implemented the proposed method DeepInterAware; Y.X. and Z.W. evaluated the performance of DeepInterAware and compared it to competing methods; F.H. carried out the theoretical analysis; W.Z., Y.X., Z.W., F.H., Z.X., Y.W., and M.Q. drafted the manuscript. W.Z. was responsible for conceptualization. All authors edited the manuscript and approved its content.

## Supporting information

Supporting Information

## Data Availability

The data that support the findings of this study are available in the supplementary material of this article.

## References

[1] A. M. Scott , J. D. Wolchok , L. J. Old , Nat. Rev. Cancer 2012, 12, 278. 22437872 10.1038/nrc3236

[2] J. Chen , K. Gao , R. Wang , D. D. Nguyen , G.‐W. Wei , Annu. Rev. Biophys. 2021, 50, 1. 33064571 10.1146/annurev-biophys-062920-063711 PMC8155790

[3] I. A. Wilson , R. L. Stanfield , Curr. Opin. Struct. Biol. 1993, 3, 113.

[4] R. D. Grange , J. P. Thompson , D. G. Lambert , Br. J. Anaesth. 2014, 112, 213. 24431350 10.1093/bja/aet293

[5] C. Schneider , A. Buchanan , B. Taddese , C. M. Deane , Bioinformatics 2022, 38, 377. 34546288 10.1093/bioinformatics/btab660 PMC8723137

[6] D. M. Mason , S. Friedensohn , C. R. Weber , C. Jordi , B. Wagner , S. M. Meng , R. A. Ehling , L. Bonati , J. Dahinden , P. Gainza , B. E. Correia , S. T. Reddy , Nat. Biomed. Eng. 2021, 5, 600. 33859386 10.1038/s41551-021-00699-9

[7] Y. W. Lim , A. S. Adler , D. S. Johnson , vol. 14, ISSN 1942‐0862, 2022, pp. 2069075.

[8] Y. Huang , Z. Zhang , Y. Zhou , Front. Immunol. 2022, 13, 1053617. 36618397 10.3389/fimmu.2022.1053617 PMC9813736

[9] J. Zhang , Y. Du , P. Zhou , J. Ding , S. Xia , Q. Wang , F. Chen , M. Zhou , X. Zhang , W. Wang , H. Wu , L. Lu , S. Zhang , Nat. Mach. Intell. 2022, 4, 964.

[10] Z. Lin , H. Akin , R. Rao , B. Hie , Z. Zhu , W. Lu , N. Smetanin , R. Verkuil , O. Kabeli , Y. Shmueli , A. dos Santos Costa , M. Fazel‐Zarandi , T. Sercu , S. Candido , A. Rives , Science 2023, 379, 1123. 36927031 10.1126/science.ade2574

[11] A. Elnaggar , M. Heinzinger , C. Dallago , G. Rihawi , Y. Wang , L. Jones , T. Gibbs , T. Feher , C. Angerer , M. Steinegger , D. Bhowmik , B. Rost , arXiv preprint arXiv:2007.06225 2007. 10.1109/TPAMI.2021.3095381 34232869

[12] N. Wang , J. Bian , Y. Li , X. Li , S. Mumtaz , L. Kong , H. Xiong , 6, 548.

[13] B. Shao , J. Yan , Nat. Commun. 2024, 15, 9392. 39477977 10.1038/s41467-024-53759-4 PMC11525655

[14] J. N. Clifford , M. H. Høie , S. Deleuran , B. Peters , M. Nielsen , P. Marcatili , Protein Science 2022, 31, e4497. 36366745 10.1002/pro.4497 PMC9679979

[15] T. Rose , N. Monti , N. Anand , T. Shen , 2024, 2024.02.08.575577.

[16] Y. Wang , Y. Xia , J. Yan , Y. Yuan , H.‐B. Shen , X. Pan , Nat. Commun. 2023, 14, 7861. 38030641 10.1038/s41467-023-43597-1 PMC10687269

[17] L. Jing , S. Xu , Y. Wang , Y. Zhou , T. Shen , Z. Ji , H. Fang , Z. Li , S. Sun , vol. 38, ISSN 2374‐3468, 2024, pp. 2661–2669.

[18] J. Zhou , B. Ji , R. Niu , X. Shang , Z. You , 304, 112549.

[19] B. E. Suzek , Y. Wang , H. Huang , P. B. McGarvey , C. H. Wu , the UniProt Consortium 31, 926. 10.1093/bioinformatics/btu739 PMC4375400 25398609

[20] T. H. Olsen , F. Boyles , C. M. Deane , 31, 141.

[21] T. H. Olsen , I. H. Moal , C. M. Deane , Bioinformatics Advances 2022, 2, vbac046. 36699403 10.1093/bioadv/vbac046 PMC9710568

[22] J. A. Ruffolo , J. J. Gray , J. Sulam , arXiv:2112.07782 2021.

[23] J. A. Ruffolo , L.‐S. Chu , S. P. Mahajan , J. J. Gray , Nat. Commun. 2023, 14, 2389. 37185622 10.1038/s41467-023-38063-x PMC10129313

[24] M. Kalemati , A. Noroozi , A. Shahbakhsh , S. Koohi , 14, 29141. 10.1038/s41598-024-80940-y PMC11589832 39587231

[25] M. Baek , 21, 1382.

[26] S. Honda , K. Koyama , K. Kotaro , in ICML 2020 workshop on computational biology (WCB) . 2020.

[27] A. Del Vecchio , A. Deac , P. Liò , P. Veličković , 2021, arXiv:2106.00757.

[28] S. Pittala , C. Bailey‐Kellogg , Bioinformatics 2020, 36, 3996. 32321157 10.1093/bioinformatics/btaa263 PMC7332568

[29] L. F. Krapp , L. A. Abriata , F. Cortés Rodriguez , M. Dal Peraro , Nat. Commun. 2023, 14, 2175. 37072397 10.1038/s41467-023-37701-8 PMC10113261

[30] Y. Tsuchiya , K. Mizuguchi , Protein Sci.: A Public. Protein Soc. 2016, 25, 815. 10.1002/pro.2874 PMC4941225 26749247

[31] D. Kuroda , H. Shirai , M. Kobori , H. Nakamura , Proteins: Struct., Funct., Bioinf. 2008, 73, 608. 10.1002/prot.22087 18473362

[32] T. Osajima , T. Hoshino , Comput. Biol. Chem. 2016, 64, 368. 27591792 10.1016/j.compbiolchem.2016.08.004

[33] L. Qu , X. Qiao , F. Qi , N. Nishida , T. Hoshino , J. Chem. Inf. Model. 2021. 10.1021/acs.jcim.1c00167 33934602

[34] Y. Kang , D. Leng , J. Guo , L. Pan , arXiv.org 2021.

[35] L. C. Xue , J. P. Rodrigues , P. L. Kastritis , A. M. Bonvin , A. Vangone , Bioinformatics 2016, 32, 3676. 27503228 10.1093/bioinformatics/btw514

[36] P. L. Kastritis , J. P. G. L. M. Rodrigues , G. E. Folkers , R. Boelens , A. M. J. J. Bonvin , J. Mol. Biol. 2014, 426, 2632. 24768922 10.1016/j.jmb.2014.04.017

[37] Y. Mo , X. Hong , B. Gao , Y. Jia , Y. Lan , arXiv:2405.17802 2024.

[38] J. Schymkowitz , J. Borg , F. Stricher , R. Nys , F. Rousseau , L. Serrano , 33, W382. 10.1093/nar/gki387 PMC1160148 15980494

[39] X. Huang , R. Pearce , Y. Zhang , 36, 1135.

[40] R. Jin , Q. Ye , J. Wang , Z. Cao , D. Jiang , T. Wang , Y. Kang , W. Xu , C.‐Y. Hsieh , T. Hou , 25, bbae304. 10.1093/bib/bbae304 PMC11221889 38960407

[41] Z. Han , F. Yang , J. Huang , C. Zhang , J. Yao , in, 2022 IEEE/CVF Conference on Computer Vision and Pattern Recognition (CVPR) , ISSN 2575‐7075, 2022, pp. 20675–20685.

[42] H. Wallach , H. Larochelle , A. Beygelzimer , F. d'Alché‐Buc , E. Fox , R. Garnett , Adv. Neural Inf. Process. Syst. 2019, 32.

[43] H. Tsuruta , H. Yamazaki , R. Maeda , R. Tamura , J. N. Wei , Z. Mariet , P. Phloyphisut , H. Shimokawa , J. R. Ledsam , L. Colwell , A. Imura , arXiv:2306.03329 2023.

[44] J. Dunbar , K. Krawczyk , J. Leem , T. Baker , A. Fuchs , G. Georges , J. Shi , C. M. Deane , Nucleic Acids Res. 2014, 42, D1140. 24214988 10.1093/nar/gkt1043 PMC3965125

[45] B. T. Foley , B. T. M. Korber , T. K. Leitner , C. Apetrei , B. Hahn , I. Mizrachi , J. Mullins , A. Rambaut , S. Wolinsky , 2018, LA‐UR‐18‐25673.

[46] M. I. J. Raybould , A. Kovaltsuk , C. Marks , C. M. Deane , Bioinformatics 2021, 37, 734. 32805021 10.1093/bioinformatics/btaa739 PMC7558925

[47] S. Sirin , J. R. Apgar , E. M. Bennett , A. E. Keating , Protein. Sci. 2016, 25, 393. 26473627 10.1002/pro.2829 PMC4815335

[48] J. Jankauskaitė, B. Jiménez‐García , J. Dapkūnas, J. Fernández‐Recio , I. H. Moal , Bioinformatics 2019, 35, 462. 30020414 10.1093/bioinformatics/bty635 PMC6361233

[49] M. Chen , C. J. T. Ju , G. Zhou , X. Chen , T. Zhang , K.‐W. Chang , C. Zaniolo , W. Wang , Bioinformatics 2019, 35, i305. 31510705 10.1093/bioinformatics/btz328 PMC6681469

[50] S. Lu , Q. Hong , B. Wang , H. Wang , IEEE Access 2020, 8, 127834.

[51] P. Bai , F. Miljković , B. John , H. Lu , Nat. Mach. Intell. 2023, 5, 126.

