# Supplementary material for: DeepInterAware: Deep Interaction Interface‐Aware Network for Improving Antigen‐Antibody Interaction Prediction from Sequence Data
Source: Adv Sci (Weinh). 2025 Feb 11;12(13):2412533. doi: 10.1002/advs.202412533 (PMC11967782; doi:10.1002/advs.202412533)
Supplement: Supplementary file 1 — Supporting Information [file ADVS-12-2412533-s001.pdf]

ADVANCED SCIENCE
Open Access

Supporting Information

for *Adv. Sci.*, DOI 10.1002/advs.202412533

DeepInterAware: Deep Interaction Interface-Aware Network for Improving Antigen-Antibody Interaction Prediction from Sequence Data

*Yuhang Xia, Zhiwei Wang, Feng Huang, Zhankun Xiong, Yongkang Wang, Minyao Qiu and Wen Zhang**

Supporting Information

Contents

1 Experiment Setup **1**
1.1 Dataset . . . 1
1.2 Baseline Methods . . . 2
1.3 Implementation . . . 4
2 Methods **4**
2.1 Sequence Encoder . . . 4
2.2 CNN-Block . . . 4
2.3 Interaction Information Pooling and AA-wise Self-attention Pooling . . . 5
2.4 Projector . . . 5
3 Supplementary Results **5**
3.1 More Results in the Transferability Experiments on CoV-AbDab Dataset . . . 5
3.2 More Results for Identifying Binding Sites . . . 5
3.3 More Results for Predicting the Binding Free Energy Changes . . . 6
3.4 More Results for Screening Potential Antibodies Binding to HER2 Target . . . 7
3.5 Ablation Study . . . 7
References **11**

1 Experiment Setup

1.1 Dataset

In this study, we used several datasets to evaluate DeepInterAware's performance and support our findings. Specifically, we employed the AVIDa-hIL6[1] and SAbDab[2] datasets for binding prediction, HIV[3] and CoV-AbDab[4] datasets for neutralization prediction. Additionally, we adopted the SAbDab dataset for binding site prediction, AB-Bind[5] and SKEMPI2[6] datasets for binding free energy change prediction. All datasets and their corresponding sources are accessible at our GitHub repository. Figure S1 illustrates the sequence homology of the antigens and antibodies in these datasets, highlighting the varied performances of different methods on various datasets.

AVIDa-hIL6 is a comprehensive Ag-Ab binding sequence dataset for predicting AAIs in the variable domain of heavy chain antibodies (VHHs), featuring the wild-type IL-6 protein and its 30 mutants as antigens. This dataset contains 20,980 labeled binding pairs and 552,911 non-binding pairs. For our study, we refined the dataset by using ANARCI to extract CDR loops from antibody sequences and removed duplicate Ag-Ab pairs with identical CDR loops. This process yielded a curated AVIDa-hIL6 dataset with 10,178 binding pairs and 315,708 non-binding pairs.

We conducted five independent experiments for the binding prediction task on the AVIDa-hIL6 dataset, each using a distinct random seed to ensure diverse dataset splits. In each split, we randomly selected 15 antigen mutants and reserved their Ag-Ab pairs for the test set, with the remaining pairs divided into training and validation sets in a 9:1 ratio. We trained the model on the training set, optimized hyperparameters on the validation set, and evaluated performance on the test set. Finally, we reported the mean and variance of results across the five experiments.

SAbDab database is a comprehensive collection of all accessible Ag-Ab complexes curated from the Protein Data Bank (PDB). From this database, we selected complexes where antigen sequences contain more than 50 amino acids, resulting in 1,193 complexes. Following the same process used for the AVIDa-hIL6 dataset, we filtered out duplicates based on antibody CDR loops, yielding a refined set of 1,513 Ag-Ab pairs. According to the splitting method of Huang et al.[7], we used CD-HIT[8] to calculate sequence homology between antigen and antibody sequences. These complexes were further divided into 772 subgroups based on antigen sequence homology, with a threshold of 0.9. Similar antibodies would bind to similar antigens within the same subgroup, whereas antigens and antibodies from different subgroups are unlikely to bind effectively. This categorization resulted in two groups: 2,131 binding pairs and an equal number of non-binding pairs. Since the sequences of antibodies binding to different antigens can vary significantly, models trained on one antigen may perform poorly when predicting interactions with substantially different antigens. To address this, we used ClustalW[9] to construct phylogenetic trees for the 772 antigen subgroups and divided them into seven clusters. This approach accounted for antigen diversity and enabled the creation of a more balanced dataset.

For the binding prediction task, we conducted five independent experiments, each using a distinct random seed to ensure diverse dataset splits. The training and independent test sets were generated from each cluster in a 4:1 ratio, ensuring that antigen type differences did not introduce bias. The model was trained on the training set, and its performance was evaluated on the test set. The mean and variance of the results from the five experiments were reported. For the binding site identification task, we performed five-fold cross-validation on 1,513 Ag-Ab pairs and reported the mean and variance of the results across the five folds.

HIV sequence database comprises neutralization antibodies related to the Human Immunodeficiency Virus (HIV). Following the protocol established by Zhang et al. [10], we filtered out Ag-Ab pairs with homology levels exceeding 0.9 for both the antigens and the antibodies. After stringent processing, the curated HIV sequence dataset comprises 24,907 neutralization pairs and 26,480 non-neutralization pairs, derived from a diverse set of 1,752 antigens and 457 antibodies.

For the neutralization prediction task on the HIV dataset, we considered three scenarios: Ab Unseen, Ag Unseen, and Ag&Ab Unseen. For each scenario, we conducted five independent experiments, each utilizing a distinct random seed to ensure the diverse dataset split. For the Ab Unseen scenario, 15% of the antibodies were randomly selected as unseen antibodies, with their corresponding Ag-Ab pairs used for the test set. The remaining pairs were divided into training and validation sets in a ratio of 9:1, ensuring that the antigens in the test set were present in the training set. In the Ag Unseen scenario, 20% of the antigens were randomly chosen as unseen antigens, and the related Ag-Ab pairs formed the test set. The rest of the pairs were also split into training and validation sets in a ratio of 9:1, with the testing antibodies ensured to be included in the training set. For the Ag&Ab Unseen scenario, building upon the Ag Unseen scenario, we removed pairs related to antibodies that appeared in the test set from the training set. The model was trained on the training set, hyperparameters were optimized using the validation set, and performance was evaluated on the test set. The mean and variance of results from the five experiments were reported.

CoV-AbDab database provides detailed information on conventional antibodies and nanobodies capable of binding to various coronaviruses. We collected the Ag-Ab neutralization and non-neutralization pairs, along with antibody sequences, from the CoV-AbDab database. Since the CoV-AbDab includes only antigen names but not sequences, we retrieved antigen sequences with annotations for the receptor-binding domain (RBD) from the database's provided references. Subsequently, we intercepted the RBD region to serve as the antigen sequence. Following the same procedure as with the AVIDa-hIL6 dataset, we have filtered out duplicates of Ag-Ab pairs. The final CoV-AbDab dataset comprises neutralization relationships between 30 coronavirus antigens and 4,235 antibodies, including 5,486 neutralization pairs and 9,110 non-neutralization pairs with sequences.

In the transferability experiment, we conducted five independent experiments using five random seeds for dataset splitting. In each split, 30% of the collected Ag-Ab pairs were used as the transfer training set, while the remaining 70% formed the test set. Highly homologous pairs with sequence similarity above 0.9 were removed, as done for the HIV dataset. The model was trained on the training set and evaluated on the test set. The mean and variance of the results from the five experiments were reported.

AB-Bind and SKEMPI2 AB-Bind database includes 1,101 mutants with experimentally determined changes in binding free energies ($\triangle\triangle G$) across 32 Ag-Ab complexes. We screened the Ag-Ab mutants annotated with both light and heavy chains, resulting in 654 mutants. SKEMPI2 database provides data on changes in protein-protein binding energy, kinetics, and thermodynamics upon mutations. As we did on the AB-Bind, we screened Ag-Ab mutants annotated with both light and heavy chains, resulting in 1,021 mutants. Following previous study [11], the dissociation constant (K_d) is used to represent the affinity, and the free energy change ($\triangle$G) can be calculated using the formula $\triangle$G = $RTln(K_d)$, where R is the ideal gas constant (1.987e-3 $kcal \cdot mol^{-1} \cdot K^{-1}$) and T ($298K$) is the absolute temperature.

For binding free energy change prediction task, we conducted ten-fold cross-validation experiments on both datasets. As in the binding site experiments, the results for each fold were recorded, and the mean and variance of the results across ten folds were reported.

1.2 Baseline Methods

For the AAI prediction, we compared DeepInterAware with several state-of-the-art methods. These include AAI prediction methods such as **DeepAAI**[10], **AbAgIntPre**[7], and **MasonCNN**[12]. Additionally, we considered several biomolecular interaction prediction methods, such as protein-protein interaction prediction methods like **PIPR**[13] and **ResPPI**[14], as well as the drug-target interaction prediction method **DrugBAN**[15], and also used pre-trained language models ESM2, AbLang and AntiBERTy to build baselines **ESM2AbLang** and **ESM2AntiBERTy**. To ensure the users can easily replicate the baselines,

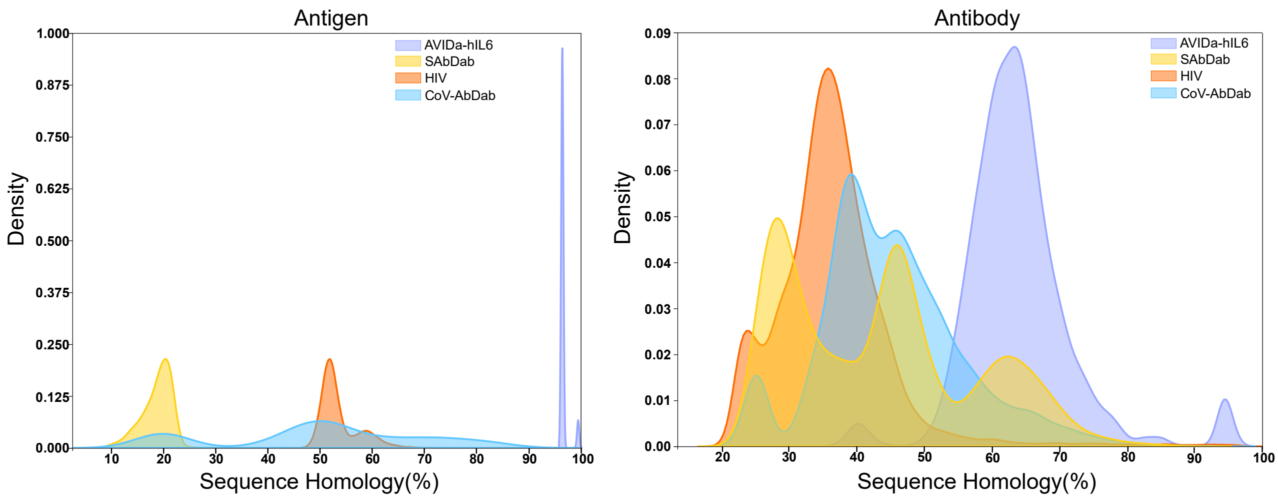

Figure S1. Sequence homology analysis of antigens and antibodies in the AAIs datasets.

we shared the source code for implementing the baselines at our GitHub repository.

DeepAAI learns the global features of antigens and antibodies from an adaptive relational graph constructed from their sequences and also utilizes CNN to capture local sequence features. By integrating global and local features, DeepAAI demonstrates exceptional performance in predicting the neutralization capacity of unseen antibodies against seen antigens. In DeepAAI, to keep the kmer feature dimensions consistent across datasets, we set the analyzed amino acid sequence length k to $k = 1,2,3$, which generates 20, 20^2 and 20^3 dimensions, respectively. Finally, an 8,420-dimensional vector of kmer is left. The remaining hyperparameters are consistent with those in the original paper.

AbAgIntPre is a deep learning-assisted prediction method for predicting the AAIs that only relies on amino acid sequences. A Siamese-like convolutional neural network architecture was established with the amino acid composition encoding scheme for both antigens and antibodies. To make this method better suited to the task of our paper, for the prediction head, we set it to be consistent with our method (a three-layer MLP). The remaining hyperparameters are consistent with those in the original paper.

MasonCNN designs a CNN to learn features from antibody sequences and builds the model that predicts antibodies binding to a specific antigen. To extend its applicability to the AAIs, we introduced a CNN to learn features from antigen sequences, which have the same architecture as the one used for antibodies. This modification aligns with the architectural approach adopted by Zhang et al[10].

PIPR seamlessly integrates a deep residual recurrent CNN within a Siamese architecture to predict protein-protein interactions, capturing both the nuanced local features and the broader contextual information embedded within protein sequences. Zhang et al.[10] have implemented an accessible code for predicting the AAIs based on this method, which we used to conduct the related experiments.

ResPPI is an efficient algorithm based on the residual network to predict protein-protein interactions. For this method, Zhang et al.[10] also provided an accessible code for predicting the AAIs, which we leveraged to perform the corresponding experiments.

DrugBAN is a deep bilinear attention network (BAN) framework with domain adaptation to explicitly learn pairwise local interactions between drugs and targets and adapt in response to out-of-distribution data. By replacing the encoders originally designed for drugs and targets with those tailored for antigens and antibodies, DrugBAN's methodological flexibility allows it to be repurposed for predicting the AAIs. The hyperparameters remain the same as in the original paper.

ESM2AbLang leverages the protein language model, ESM2[16], to extract features from antigen sequences. It also harnesses the power of the antibody-specific language model, AbLang[17], to capture features from antibody sequences. The extracted features from both models are then seamlessly integrated and fed into a downstream classifier that aligns with our method. For the pre-trained model version of ESM2, we chose esm2_t12_35M_UR50D with 12 layers and 35M parameters. For AbLang, we used the pre-trained model available in the AbLang code repository.

ESM2AntiBERTy is the variant of ESM2AbLang, which simply replaces AbLang with the antibody-specific language model AntiBERTy[18]. For AntiBERTy, we used the pre-trained model available in the AntiBERTy code repository.

For binding site identification, we evaluated state-of-the-art prediction methods, including the sequence-based method

(**Honda et al.'s method**[19]) as well as the structure-based methods **EPI-EPMP**[20], **PECAN**[21], and **PesTo**[22]. Honda proposed a cross-attention network that encodes antigen and antibody sequences, computes interaction attention matrices, and predicts binding sites using linear layers. EPI-EPMP employs a graph attention neural network (GAT) to encode the structural graphs of antigens and antibodies, utilizing graph attention matrices to predict binding sites through fully connected layers. PECAN uses graph convolutional networks (GCNs) to encode the structures of antibodies and antigens, applying interaction attention to predict binding sites. PesTo encodes atomic coordinates to obtain residue-level binding probabilities of proteins, making it suitable for predicting both paratopes and epitopes, as the antibodies and antigens in our study are proteins. We implemented these methods using source code from their original publications with default hyperparameters.

For binding free energy change prediction, we considered several state-of-the-art binding free energy change prediction methods, including the force field-based methods **FoldX** [23] and **EvoEF** [24], and deep learning-based method **AttABseq**[25]. FoldX is a standalone tool leveraging a physical force field to accurately predict changes in binding free energy for protein-protein and protein-ligand interactions. EvoEF, another standalone tool, employs a composite energy force field for binding free energy change prediction. As these tools do not require a training process, FoldX and EvoEF were directly evaluated on the test set. For the deep learning-based method AttABseq, we trained the model on the training set using the code and default hyperparameters provided in its publication, and subsequently evaluated its performance on the test set.

1.3 Implementation

DeepInterAware is implemented in Python 3.7 and PyTorch 1.12.1, along with functions from transformers 4.39.0 and torchmetrics 0.11.4. All experiments were conducted on a 24GB NVIDIA GeForce RTX 3090. The Adam optimizer was used with a learning rate of 1e-3. In the bilinear attention module, two attention heads were employed.

As described in Section 4.2.5 of the manuscript, the model training consists of two stages. For model training in the binding prediction task, we set epochs of 20 (stage 1) and 10 (stage 2), a batch size of 512 and a dropout rate of 0.5 on the AVIDa-hIL6 dataset, and epochs of 100 and 50, a batch size of 128 and a dropout rate of 0.1 on the SAbDab dataset. For model training in the neutralization prediction task, we set epochs of 30 and 20, a batch size of 256 and a dropout rate of 0.5 on the HIV dataset, and epochs of 75 and 75, batch size of 32 on the CoV-AbDab dataset. For more details on hyperparameters settings, please refer to the source code at our GitHub repository.

2 Methods

Figure S2 illustrates the architecture of some modules in DeepInterAware, which are described in detail as follows.

2.1 Sequence Encoder

DeepInterAware is built upon the foundation of the protein language model ESM2[16] and the antibody language model AbLang[17]. Formally, for an Ag-Ab pair $\{x_{Ag}, x_{Ab}\}$, we obtain their initial amino acid features by Equations (1) and (2),

$$\mathbf{X}_{Ag} = \text{ESM2}(x_{Ag}), \tag{1}$$

$$\mathbf{X}_{Ab} = \text{AbLangH}(x^H_{Ab}) \oplus \text{AbLangL}(x^L_{Ab}) \tag{2}$$

where $\mathbf{X}_{Ag} \in \mathbb{R}^{M \times d_1} (d_1 = 480)$ and $\mathbf{X}_{Ab} \in \mathbb{R}^{N \times d_2} (d_2 = 768)$ are representations of the amino acids in each antigen and antibody sequence, and M and N denote the number of amino acids.

2.2 CNN-Block

CNN-Block conducts 1D convolution on the amino acid representations $\mathbf{X}_{Ag}$ and $\mathbf{X}_{Ab}$, in which the channels, kernel size, stride, and padding are 128 (192), 3, 1, and 1, respectively. The maximum pooling in which the kernel size, stride, and padding are 3, 1, and 1. The complete CNN-Block architecture is shown in Figure S2c.

$$\mathbf{H}^{(1)} = \sigma(\text{CNN}(\mathbf{W}_c, \mathbf{b}_c, \mathbf{X})), \tag{3}$$

$$\mathbf{H}^{(2)} = \text{BatchNorm}(\mathbf{H}^{(1)}), \tag{4}$$

$$\mathbf{H} = \text{MaxPool}(\mathbf{H}^{(2)}) \tag{5}$$

where $\mathbf{X}$ represents $\mathbf{X}_{Ag}$ or $\mathbf{X}_{Ab}$. $\mathbf{W}_c$ and $\mathbf{b}_c$ are the learnable weight matrices (filters) and bias vectors in the CNN layer. $\sigma(\cdot)$ denotes a non-linear activation function, with ReLU.

2.3 Interaction Information Pooling and AA-wise Self-attention Pooling

Interaction Information Pooling (IIP) and AA-wise Self-attention Pooling (ASP) have similar structures, both centered around the Activation Unit, as shown in Figure S2a and S2b. As shown in Figure S2d, the Activation Unit computes amino acid-level feature weights from the input features, normalizes these weights using the softmax function, and performs sum pooling based on these weights to obtain sequence-level features. The key difference between IIP and ASP lies in the basis for weight calculation: IIP uses interaction information to compute amino acid weights, enhancing the modeling of AAIs, while ASP derives weights directly from the amino acid features, focusing on the intrinsic properties of individual amino acids.

2.4 Projector

The projector is used for the initial fusion of concatenated Ag-Ab features, projecting interaction features and specificity features into the same space for subsequent Dynamic Confidence Fusion, as shown in Figure S2e.

3 Supplementary Results

3.1 More Results in the Transferability Experiments on CoV-AbDab Dataset

Table S1 shows the transferability performance of all methods in five independent experiments. The results demonstrate DeepInterAware's exceptional transferability capacity.

Table S1. Transferability performance comparison on CoV-AbDab dataset.

Model	AUROC	AUPRC	MCC	ACC	F1	Precision	Recall
ESM2AbLang	0.792±0.005	0.684±0.008	0.442±0.008	0.737±0.006	0.654±0.007	0.653±0.017	0.656±0.027
ESM2AntiBERTy	0.791±0.008	0.682±0.007	0.437±0.010	0.733±0.004	0.654±0.007	0.637±0.005	0.672±0.012
ResPPI	0.769±0.016	0.677±0.018	0.383±0.011	0.717±0.010	0.562±0.041	0.694±0.068	0.486±0.098
PIPR	0.775±0.002	0.679±0.003	0.389±0.007	0.689±0.005	0.645±0.003	0.569±0.007	<u>0.746±0.010</u>
DrugBAN	0.813±0.004	0.735±0.006	<u>0.511±0.008</u>	<u>0.774±0.004</u>	<u>0.683±0.007</u>	<u>0.730±0.014</u>	0.641±0.018
AbAgIntPre	0.812±0.003	0.727±0.005	0.464±0.011	0.745±0.008	0.670±0.012	0.660±0.024	0.683±0.045
MasonsCNN	**0.842±0.009**	<u>0.749±0.013</u>	0.510±0.029	0.747±0.025	**0.711±0.015**	0.625±0.035	**0.827±0.032**
DeepAAI	0.800±0.035	0.710±0.037	0.435±0.053	0.738±0.025	0.620±0.060	0.696±0.073	0.576±0.113
DeepInterAware	<u>0.838±0.006</u>	**0.758±0.016**	**0.537±0.017**	**0.787±0.007**	0.680±0.028	**0.783±0.031**	0.605±0.058

The best results are marked in **bold** and the second-best results are <u>underlined</u>. - represents that the method is unsuitable for the current task.

3.2 More Results for Identifying Binding Sites

In this study, we followed the conventional definitions of epitopes and paratopes. Specifically, we utilized Euclidean distance as the metric. A residue in the CDR regions or antigen is labeled as a paratope or epitope if the Euclidean distance between its backbone atom and any backbone atom of the counterpart antigen or CDR regions is less than 5.0 Å. In DeepInterAware, potential binding sites are identified by averaging the attention map across the corresponding dimensions of both antigens and antibodies. This process calculates attention weights for amino acids in both antigens and antibodies. These weights are normalized and transformed to values between 0 and 1 using the sigmoid activation function. Residues with values exceeding a threshold of 0.5 are predicted as potential binding sites. For identifying epitope-paratope binding pairs, the attention map is normalized and converted to values between 0 and 1 using the sigmoid activation function on antibody dimensions. The residue pairs with attention scores exceeding the threshold of 0.5 are predicted as potential epitope-paratope binding pairs. To validate the binding site identification capability, we introduced a variant of DeepInterAware, termed DeepInterAware*, which uses annotated binding sites for model training, as typical binding site prediction methods do. Specifically, within the DeepInterAware's framework shown in Figure 1 of the manuscript, we removed the IIP and ASP modules responsible for learning sequence-level features, and instead directly employed the DCF module to integrate the amino acid-level features extracted by the IIL and SIL learners for antigens and antibodies. For binding site identification, the features of each residue on the antibody and antigen, derived from the DCF module, are independently fed into MLP-based predictors. These predictors estimate the probability of a residue being the binding site.

To ensure a fair comparison, we used the SAbDab dataset and performed five-fold cross-validation to evaluate our method alongside state-of-the-art binding site prediction methods. The AUROC and AUPRC scores for all methods are summarized in Table S2. Although DeepInterAware performs much poorer than the binding site prediction methods, its variant DeepInter-Aware* can deliver the best or second-best results among all methods, demonstrating that DeepInterAware can be extended to achieve even greater prediction accuracy when annotated binding sites are used during model training.

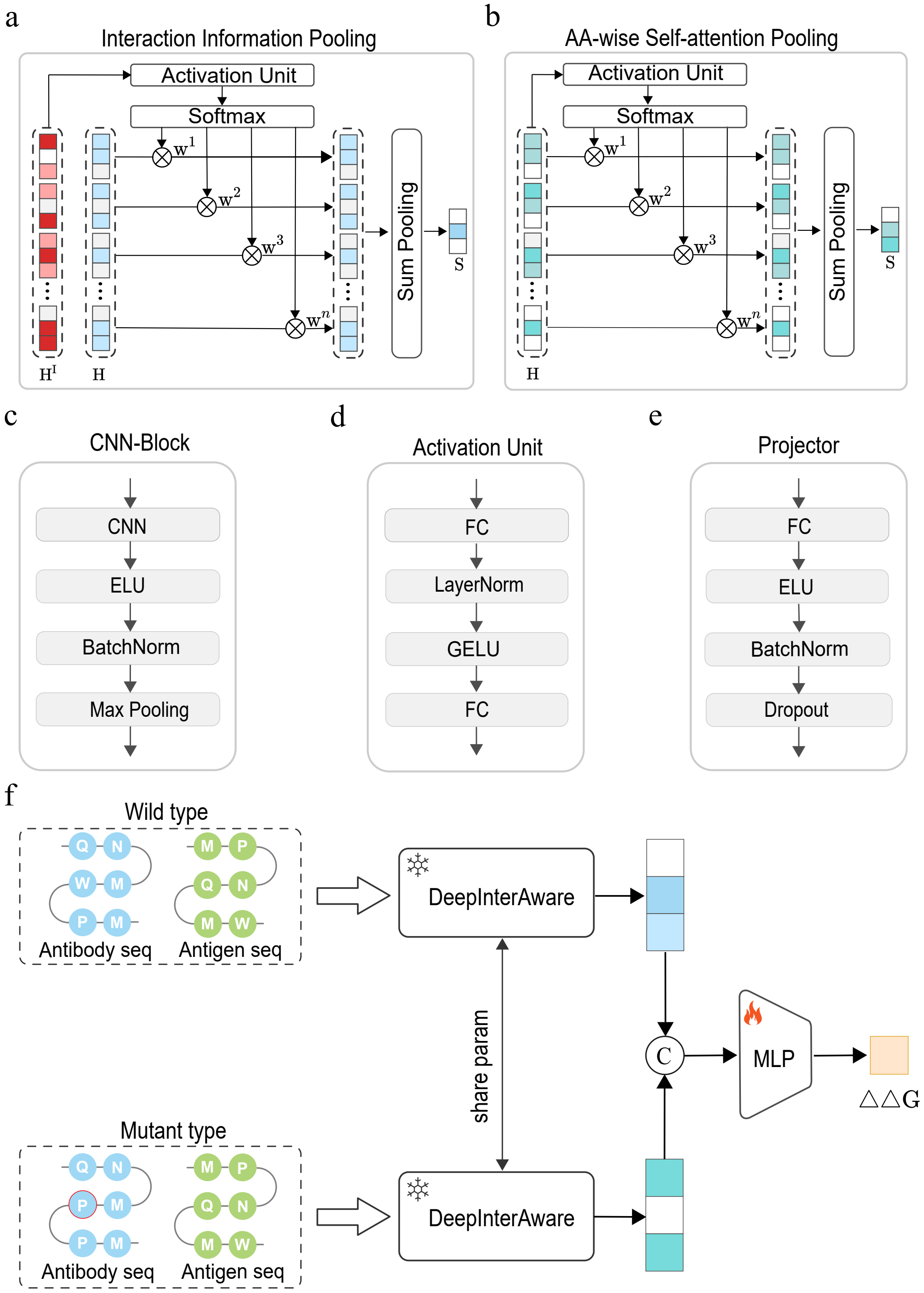

Figure S2. The architecture of some modules in DeepInterAware. **a**.Interaction Information Pooling framework. **b**.AA-wise Self-attention Pooling framework. **c**. CNN-Block framework. **d**. Activation Unit framework. **e**.Projector framework. **f**.The Workflow of the extended DeepInterAware on antigen-antibody binding free energy change prediction task.

3.3 More Results for Predicting the Binding Free Energy Changes

To evaluate the performance of DeepInterAware in predicting binding free energy changes upon mutations, we extended DeepInterAware's architecture, as illustrated in Figure S2f. We utilized the Ag-Ab binding model as a feature extractor for Ag-Ab pairs, froze its parameters, and constructed a three-layer MLP to map the concatenated wild-type and mutant pair

Table S2. Binding site identification performance comparison on SAbDab dataset.

Model	Antigen		Antibody	
	AUROC	AUPRC	AUROC	AUPRC
PesTo	0.771±0.151	**0.530±0.104**	0.765±0.130	0.626±0.104
PECAN	0.648±0.152	0.289±0.140	0.801±0.149	0.518±0.124
EPI-EPMP	**0.824±0.1433**	0.308±0.248	0.843±0.127	0.663±0.185
Honda	0.594±0.171	0.266±0.166	<u>0.863±0.133</u>	<u>0.679±0.113</u>
**DeepInterAware**	0.635±0.091	0.203±0.124	0.637±0.081	0.434±0.113
**DeepInterAware***	<u>0.781±0.018</u>	<u>0.437±0.025</u>	**0.898±0.007**	**0.742±0.019**

The best results are marked in **bold** and the second-best results are <u>underlined</u>. - represents that the method is unsuitable for the current task.

features into binding free energy changes. We used AB-Bind and SKEMPI2 datasets to evaluate DeepInterAware's capabilities in binding free energy change prediction. Given the presence of mutations outside the CDR regions in these datasets, full-length antibody sequences, rather than only the CDR regions, were used during model training. We compared the extended DeepInterAware, with some binding free energy change prediction methods, including FoldX [23], EvoEF [24], and AttABseq [25], and evaluated these methods by ten-fold cross-validation on the AB-Bind and SKEMPI2 datasets. As shown in Table S3, the extended DeepInterAware outperforms these methods in terms of all metrics (PCC, RMSE, and MSE). Compared to the second-best method AttABseq, our extended method demonstrates relative improvements of 4.6%, 15.7%, and 22.4% in PCC, RMSE, and MAE, respectively, on the AB-Bind dataset, and 8.0%, 11.9%, and 4.0% on the SKEMPI2 dataset, demonstrating its capability in predicting binding free energy changes.

Table S3. Binding free energy change prediction performance comparison on AB-Bind and SKEMPI2 datasets.

Dataset	Model	PCC	RMSE	MAE
AB-Bind	EvoEF	0.308	2.659	1.660
	FoldX	0.354	3.499	2.142
	AttABseq	<u>0.565</u>	<u>2.169</u>	<u>1.596</u>
	**DeepInterAware**	**0.611**	**2.012**	**1.372**
SKEMPI2	EvoEF	0.315	2.001	1.443
	FoldX	0.023	3.917	2.434
	AttABseq	<u>0.445</u>	<u>1.648</u>	<u>1.157</u>
	**DeepInterAware**	**0.525**	**1.529**	**1.117**

The best results are marked in **bold** and the second-best results are <u>underlined</u>. - represents that the method is unsuitable for the current task.

3.4 More Results for Screening Potential Antibodies Binding to HER2 Target

To test the potential of DeepInterAware in virtual screening, we utilized the proposed method to screen potential antibodies targeting HER2. Table S4 summarizes the docking scores of complexes formed by screened candidates and HER2. Notably, the binding region between the candidate antibody NabFab (PDB: 7RTH) and HER2 closely resembles that of the cancer-specific therapeutic antibody H2Mab-119. Figure S3 illustrates a detailed alignment of the binding regions of HER2 when bound to H2Mab-119 and NabFab, respectively, showing 85% amino acid residue overlap. This finding highlights the remarkable similarity in the recognition of the HER2 antigen by both antibodies.

3.5 Ablation Study

To evaluate the superiority and effectiveness of critical modules in DeepInterAware, we considered several variants of DeepInterAware:

- **DeepInterAware(w/o SIL)** removes the Specificity Information Learner.
- **DeepInterAware(w/o IIL)** removes the Interaction Interface-aware Learner.
- **DeepInterAware(w/o IIP)** removes the Interaction Information Pooling module directly obtained Ag-Ab pair features by pooling from the interaction matrix using bilinear pooling.
- **DeepInterAware(w/o DCF)** replaces the Dynamic Confidence Fusion module with the concatenation operation.

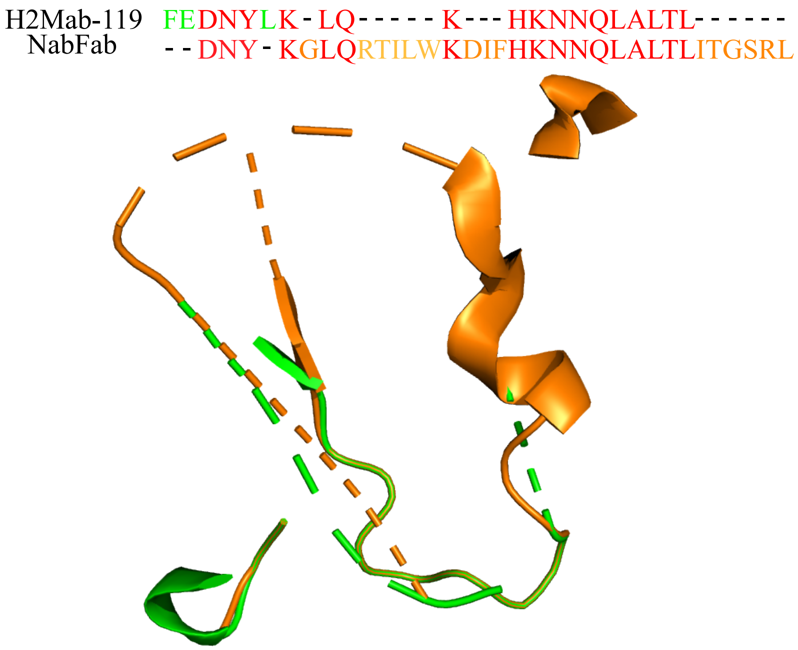

Figure S3. The alignment of binding regions of HER2 when it binds to H2Mab-119 and NabFab, respectively. The amino acids highlighted in red are those where the two regions overlap.

Table S4. Docking scores of complexes formed by screened antibodies and HER2.

PDB ID	Heavy	Light	Docking Score	PDB ID	Heavy	Light	Docking Score
4M1C	C	D	1905.228	7KGU	D	C	1650.450
8J7E	A	B	1858.725	5GS0	X	Y	1648.933
6CW2	A	B	1837.381	6OT0	H	L	1643.253
7LO7	H	L	1777.815	6MY4	A	B	1642.612
4I18	H	L	1777.524	7KEO	E	F	1639.387
7USL	H	L	1761.724	7RTH	F	E	1634.635
8T9Y	H	L	1751.250	7RTH	L	K	1629.649
4IOF	E	F	1727.336	3EFF	B	A	1627.218
2VWE	E	C	1716.320	4AG4	H	L	1626.045
8RQF	H	L	1715.746	2QQL	H	L	1617.647
7LO8	H	L	1700.171	3CXD	H	L	1617.338
7LR3	A	B	1694.745	7SRS	H	L	1611.405
7NIW	C	B	1694.379	5KEN	G	H	1610.034
4IOF	C	D	1686.609	7RTH	D	C	1609.902
8VZO	B	D	1683.108	5CJO	H	L	1609.386
5WOB	U	V	1675.253	7C61	H	L	1606.881
7RTH	H	G	1674.473	1ZA3	H	L	1606.266
5CZV	H	L	1668.851	1OPG	H	L	1606.097
7KLG	I	M	1665.963	7RTH	B	A	1605.772
4R9Y	N	M	1659.832	4KHX	H	L	1603.386
6MY5	A	B	1659.104	3PJS	D	C	1600.390
7MFG	E	F	1652.838				

- **DeepInterAware(w/o DCF*)** utilizes dynamic confidence to fuse the decision of interaction features and specific features as the model's prediction result, rather than performing feature fusion.

The performance of DeepInterAware and its variants were evaluated on the SAbDab dataset for binding prediction, and HIV

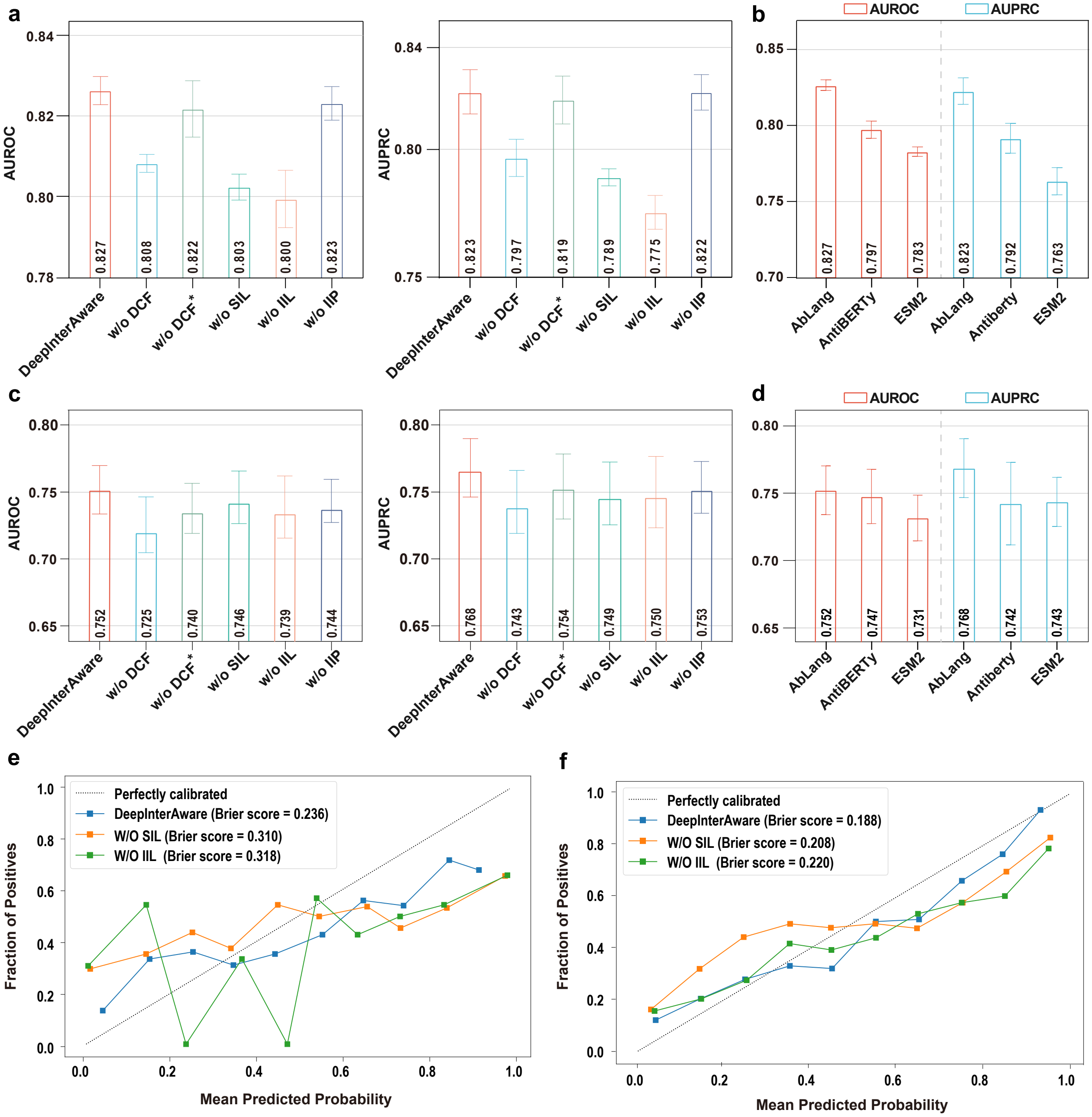

Figure S4. Ablation study. **a** and **c** show the ablation experiments of DeepInterAware and its variants on SAbDab and HIV datasets, respectively. **b** and **d** show the experimental results on SAbDab and HIV datasets using different large language models to encode antibody features, respectively. **e** and **f** display the calibration curves on the SAbDab and HIV datasets, respectively.

dataset for neutralization prediction (Ag&Ab Unseen scenario). The results are presented in Figure S4.

Overall, DeepInterAware consistently produces superior predictive performance, with the removal of any module causing noticeable performance degradation. Excluding the IIL module (w/o IIL) led to a significant drop in AUROC and AUPRC scores on the SAbDab dataset, demonstrating that IIL effectively captures physicochemical interactions between antigens and antibodies, thereby improving the models transferability in AAI prediction. Similarly, excluding the DCF module (w/o DCF) caused a notable decline in performance metrics on the HIV dataset. Results for the variant without SIL (w/o SIL) highlight the importance of incorporating SIL to improve the models predictive accuracy. Additionally, results for the variants without IIP (w/o IIP) or DCF* (w/o DCF*) underscore the critical role of pooling interaction information in enhancing the models predictive performance.

Calibration evaluates the difference between predicted probabilities and ground truth, reflecting the models accuracy and

reliability. A calibration curve closer to the diagonal indicates better agreement between predicted scores and actual results. The Brier score quantitatively measures calibration effectiveness, with lower values indicating better alignment. Figure S4e and Figure S4f show that DeepInterAware's calibration curve aligns more closely with the diagonal compared to its variants, achieving the lowest Brier score. These findings indicate that DeepInterAware provides reliable predictions consistent with real-world outcomes.

To assess the impact of different encoders on antibody feature representation, we replaced AbLang with AntiBERTy and ESM2 in DeepInterAware. The performances of DeepInterAware and its variants with different antibody encoders are presented in Figure S4b and Figure S4d. The results indicate that using ESM2 as the antibody encoder generally results in inferior performance compared to using AbLang or AntiBERTy. This difference is likely because AbLang and AntiBERTy, trained on extensive antibody-specific sequences, capture more specialized antibody knowledge than the general protein language model ESM2. Among the two antibody language models, AbLang demonstrates superior performance, likely due to its separate encoding of heavy and light chains, a strategy proven effective by Wang et al.[26].

References

[1] H. Tsuruta, H. Yamazaki, R. Maeda, R. Tamura, J. N. Wei, Z. Mariet, P. Phloyphisut, H. Shimokawa, J. R. Ledsam, L. Colwell, A. Imura **2023**, , arXiv:2306.03329.

[2] J. Dunbar, K. Krawczyk, J. Leem, T. Baker, A. Fuchs, G. Georges, J. Shi, C. M. Deane, *Nucleic Acids Research* **2014**, *42*, D1 D1140.

[3] B. T. Foley, B. T. M. Korber, T. K. Leitner, C. Apetrei, B. Hahn, I. Mizrachi, J. Mullins, A. Rambaut, S. Wolinsky **2018**, , LA-UR-18-25673.

[4] M. I. J. Raybould, A. Kovaltsuk, C. Marks, C. M. Deane, *Bioinformatics* **2021**, *37*, 5 734.

[5] S. Sirin, J. R. Apgar, E. M. Bennett, A. E. Keating, *Protein Science : A Publication of the Protein Society* **2016**, *25*, 2 393.

[6] J. Jankauskaitė, B. Jiménez-García, J. Dapkūnas, J. Fernández-Recio, I. H. Moal, *Bioinformatics* **2019**, *35*, 3 462.

[7] Y. Huang, Z. Zhang, Y. Zhou, *Frontiers in Immunology* **2022**, *13* 1053617.

[8] L. Fu, B. Niu, Z. Zhu, S. Wu, W. Li, *Bioinformatics* **2012**, *28*, 23 3150.

[9] J. D. Thompson, D. G. Higgins, T. J. Gibson, *Nucleic Acids Research* **1994**, *22*, 22 4673.

[10] J. Zhang, Y. Du, P. Zhou, J. Ding, S. Xia, Q. Wang, F. Chen, M. Zhou, X. Zhang, W. Wang, H. Wu, L. Lu, S. Zhang, *Nature Machine Intelligence* **2022**, *4*, 11 964.

[11] G. Wang, X. Liu, K. Wang, Y. Gao, G. Li, D. T. Baptista-Hon, X. H. Yang, K. Xue, W. H. Tai, Z. Jiang, L. Cheng, M. Fok, J. Y.-N. Lau, S. Yang, L. Lu, P. Zhang, K. Zhang, *Nature Medicine* **2023**, *29*, 8 2007.

[12] D. M. Mason, S. Friedensohn, C. R. Weber, C. Jordi, B. Wagner, S. M. Meng, R. A. Ehling, L. Bonati, J. Dahinden, P. Gainza, B. E. Correia, S. T. Reddy, *Nature Biomedical Engineering* **2021**, *5*, 6 600.

[13] M. Chen, C. J. T. Ju, G. Zhou, X. Chen, T. Zhang, K.-W. Chang, C. Zaniolo, W. Wang, *Bioinformatics* **2019**, *35*, 14 i305.

[14] S. Lu, Q. Hong, B. Wang, H. Wang, *IEEE Access* **2020**, *8* 127834.

[15] P. Bai, F. Miljković, B. John, H. Lu, *Nature Machine Intelligence* **2023**, *5*, 2 126.

[16] Z. Lin, H. Akin, R. Rao, B. Hie, Z. Zhu, W. Lu, N. Smetanin, R. Verkuil, O. Kabeli, Y. Shmueli, A. dos Santos Costa, M. Fazel-Zarandi, T. Sercu, S. Candido, A. Rives, *Science* **2023**, *379*, 6637 1123.

[17] T. H. Olsen, I. H. Moal, C. M. Deane, *Bioinformatics Advances* **2022**, *2*, 1 vbac046.

[18] J. A. Ruffolo, J. J. Gray, J. Sulam **2021**, , arXiv:2112.07782.

[19] S. Honda, K. Koyama, K. Kotaro, In *ICML 2020 workshop on computational biology (WCB)*. **2020** .

[20] A. Del Vecchio, A. Deac, P. Liò, P. Veličković **2021**, , arXiv:2106.00757.

[21] S. Pittala, C. Bailey-Kellogg, *Bioinformatics* **2020**, *36*, 13 3996.

[22] L. F. Krapp, L. A. Abriata, F. Cortés Rodriguez, M. Dal Peraro, *Nature Communications* **2023**, *14*, 1 2175.

[23] J. Schymkowitz, J. Borg, F. Stricher, R. Nys, F. Rousseau, L. Serrano *33* W382.

[24] X. Huang, R. Pearce, Y. Zhang *36*, 4 1135.

[25] R. Jin, Q. Ye, J. Wang, Z. Cao, D. Jiang, T. Wang, Y. Kang, W. Xu, C.-Y. Hsieh, T. Hou *25*, 4 bbae304.

[26] D. Wang, F. Ye, H. Zhou, On Pre-trained Language Models for Antibody, URL http://arxiv.org/abs/2301.12112.
